# Reference Curves for Metabolic Syndrome Indicators in Children and Adolescents: A Global Systematic Review

**DOI:** 10.1007/s13679-025-00679-z

**Published:** 2026-01-05

**Authors:** Khalid Iqbal, Ermioni Chatziangelousi, Maike Wolters, Timm Intemann, Katharina Englert, Antje Hebestreit, Krasimira Aleksandrova

**Affiliations:** 1 Department of Epidemiological Methods and Etiological Research, Leibniz Institute for Prevention Research and Epidemiology – BIPS, Achterstraße 30, 28359 Bremen, Germany; 2 Department of Research Data Infrastructures and Data Science, Leibniz Institute for Prevention Research and Epidemiology – BIPS, Bremen, Germany; 3 Faculty of Human and Health Sciences, University of Bremen, Bremen, Germany

**Keywords:** Reference curves, Metabolic syndrome, Abdominal obesity, Dyslipidaemia, Hypertension, Impaired glucose metabolism, Paediatric population

## Abstract

**Purpose of Review:**

We aimed to summarise recent evidence on age- and sex-specific reference curves for metabolic syndrome (MetS) indicators in paediatric populations.

**Recent Findings:**

There is a lack of consensus regarding diagnostic thresholds for MetS in children and adolescents, leading to challenges in its early identification and intervention.

**Summary:**

A systematic search was performed in PubMed/Medline, Web of Science and Scopus, covering the period between January 2018 and February 2025. Three researchers evaluated 8,529 studies according to the inclusion criteria. Finally, 46 articles that reported reference values for at least one metabolic indicator: waist circumference, fasting glucose, glycated haemoglobin, homeostatic model assessment for insulin resistance, high-density lipoprotein cholesterol, triglycerides, systolic or diastolic blood pressure, in children aged 0 to 18 years were included in the review and data synthesis. The age-specific trends in each MetS indicator were assessed by calculating the median reference curves along with the lower and upper percentile bounds. Overall, there has been a substantial heterogeneity in the reported reference values for waist circumference and glucose metabolism biomarkers. Comparatively smaller variations were observed for blood pressure and lipid parameters. Limited data were available for young age groups (0–4 years) and there have been substantial differences in study methodologies including study design, assays and statistical approaches used to derive reference curves. This systematic review highlighted the substantial inconsistencies in the reported reference curves for MetS indicators in children and adolescents. There is a pressing need for deriving harmonized reference curves for paediatric MetS from diverse populations.

**Supplementary Information:**

The online version contains supplementary material available at 10.1007/s13679-025-00679-z.

## Introduction

Metabolic Syndrome (MetS) typically emerges in early life and is strongly linked to an increased risk of developing various chronic diseases throughout a person’s life [1]. MetS is characterized by a cluster of conditions including abdominal obesity, high blood pressure, high blood triglycerides, low levels of high-density lipoprotein cholesterol (HDL-C), and insulin resistance. The increasing prevalence of MetS and related chronic diseases, particularly among younger populations, poses a significant public health challenge. Early identification and intervention are therefore crucial for preventing and managing MetS and its long-term consequences.

A number of paediatric MetS classifications have been suggested previously, proposing a variety of metabolic indicators and cut-off points [2–6]. Among these, the definition of MetS proposed in 2014 by investigators of the Identification and prevention of dietary- and lifestyle-induced health effects in children and infants (IDEFICS) study provided age- and sex-specific (and height-specific in the case of blood pressure) percentiles to identify cut-offs for the components of MetS in children aged 2–11 years [5]. According to the IDEFICS definition, children would require close monitoring if three or more of the metabolic indicators exceed the 90th percentile (or ≤ 10th percentile for HDL-C) whereas intervention would be deemed appropriate if three or more of metabolic indicators exceed the 95th percentile (or ≤ 5th percentile for HDL-C). This definition has been proposed for worldwide use [7] to resolve the currently missing consensus on specific cut-off values for the individual components of MetS in children and adolescents.

 Past research has emphasized the need for age- and sex- specific cut-off points and percentiles defining abdominal obesity, dyslipidaemia, elevated BP, and impaired glucose metabolism into account [2, 8–10]. Multiple studies have been published reporting on paediatric reference curves for MetS components [11–13], but still a systematic review of these newly proposed reference curves is lacking.

In order to facilitate the consensus that allows for early diagnosis, effective clinical decision-making, monitoring of changes and prevention efforts, this systematic review aimed to identify reference curves/values for metabolic indicators in relation to MetS in children and adolescents. It further aimed to explore potential differences in the reference values denoting early life metabolic risk by age and sex using the IDEFICS study definition as a reference [5].

## Materials and methods

This systematic review was conducted in line with the Preferred Reporting Items for Systematic Reviews and Meta-Analyses (PRISMA) guidelines [14]. The protocol for this systematic review was published in PROSPERO (No CRD420251089483).

### Search Strategy

Three independent researchers (EC, KE and KI) conducted a comprehensive search for scientific articles in the following electronic databases (i) PubMed by National Library of Medicine (MEDLINE) (ii) Web of Science and (iii) Scopus by Elsevier. The search was limited to studies that were published in English between January 2018 and February 2025. The population of interest included children and adolescents, with no restriction on age in years to identify maximum studies. The systematic search strategy was organized according to three search blocks depicting terms describing paediatric population, the metabolic components, and reference curves. The keywords and MeSH terms included in the search strategy are provided in the Supplementary Material (Table S1).

A total of 8,529 articles were retrieved, and their titles were checked for duplications and relevance to the review topic. Duplicate references were removed using EndNote (Version 20.2) [15]. Subsequently, the retrieved articles were exported to Covidence (Release 2022; Veritas Health Innovation Ltd, Melbourne, Australia) and screened using the inclusion and exclusion criteria to check their eligibility [16].

## Inclusion Criteria

The review questions were defined as follows: (1) What are the metabolic indicators used in the development of reference curves in relation to early life MetS in children and adolescents? (2) Which reference curves exist for individual metabolic indicators? (3) Are there differences in reported reference values denoting early life metabolic risk by age and sex. Thus, articles were considered eligible if they met the following inclusion criteria: (a) based on human participants aged 0 to 18 years (studies with participants older than 18 years were included if the study included the target age range), (b) published in English language, (c) reported age-, and sex-specific percentile values for at least one MetS indicator, i.e. waist circumference (WC), fasting glucose (FG), glycated haemoglobin (HbA1c), homeostatic model assessment for insulin resistance (HOMA-IR), HDL-C, low-density lipoprotein cholesterol (LDL-C), TG, systolic blood pressure (SBP) and diastolic blood pressure (DBP), (d) were peer-reviewed observational studies (i.e. cohort or cross-sectional studies).

## Exclusion Criteria

Studies were excluded if they: (a) reported only median or z-scores, fixed cut-off values or reference percentiles values for broader age ranges e.g. 0–5 or 6–10 years (b) were based on clinical (hospital-based) or patient populations or conducted exclusively in athlete populations (to ensure that reference values for apparently healthy pediatric population are identified, (c) conducted exclusively in athlete populations, (d) meta-analyses, systematic reviews, literature reviews, letters to the editor, and conference abstracts, (e) had missing information (age, sex and location), unclear data, or were unavailable in full text.

## Quality/Risk of Bias Assessment

The risk of bias of individual studies was assessed using an adapted version of the BIOCROSS tool, that was specifically developed for the purpose of evaluating the reporting quality of epidemiological studies utilizing biomarker data [17]. The tool encompasses five domains: ‘Study rationale’, ‘Design/Methods’, ‘Data analysis’, ‘Data interpretation’, and ‘Biomarker measurement’, assessed using a 10-item scale. In cases of disagreement, a third reviewer (MW or KI) was consulted to reach a final decision.

## Data Extraction

Data extraction of selected studies was performed using a prespecified form prepared by the research team. Two reviewers (EC and KE) independently added the extracted data to the file which was then compared to ensure accuracy. The following information was extracted: author names, publication year, study year, country where the research was conducted, study design, sample size, age of participants, sex of the participants, data collection period, and method of reference value estimation. Finally, another researcher (KI) reviewed the extracted data for verification. Articles selected based on the abstracts screening underwent full text review, and only those meeting all eligibility criteria were included. In cases of disagreement between the researchers, a third researcher (KI) made the final decision. References of the included studies were manually searched to identify additional studies.

### Data Synthesis

The characteristics of the included studies were summarized descriptively. For comparability, the reference curves of all studies for the single markers were overlaid in a single plot. To assess the overall age-specific trends in these biomarkers, the median reference curves, along with the lower and upper bounds of the 5th, 10th, 50th, 90th, and 95th percentiles from all studies, are presented. The IDEFICS study definition of MetS components proposed by Ahrens et al. [5] were overlaid on the corresponding percentiles of each component for comparison as reference. In order to clearly display the more than 24 waist circumference curves, additional interval bands (covering 50% and 100% curves) and mean values of all studies available for the respective age were derived and displayed. For this purpose, the curves from the individual studies were interpolated to cover all age values in increments of 0.25 years.

## Results

**Fig. 1:**
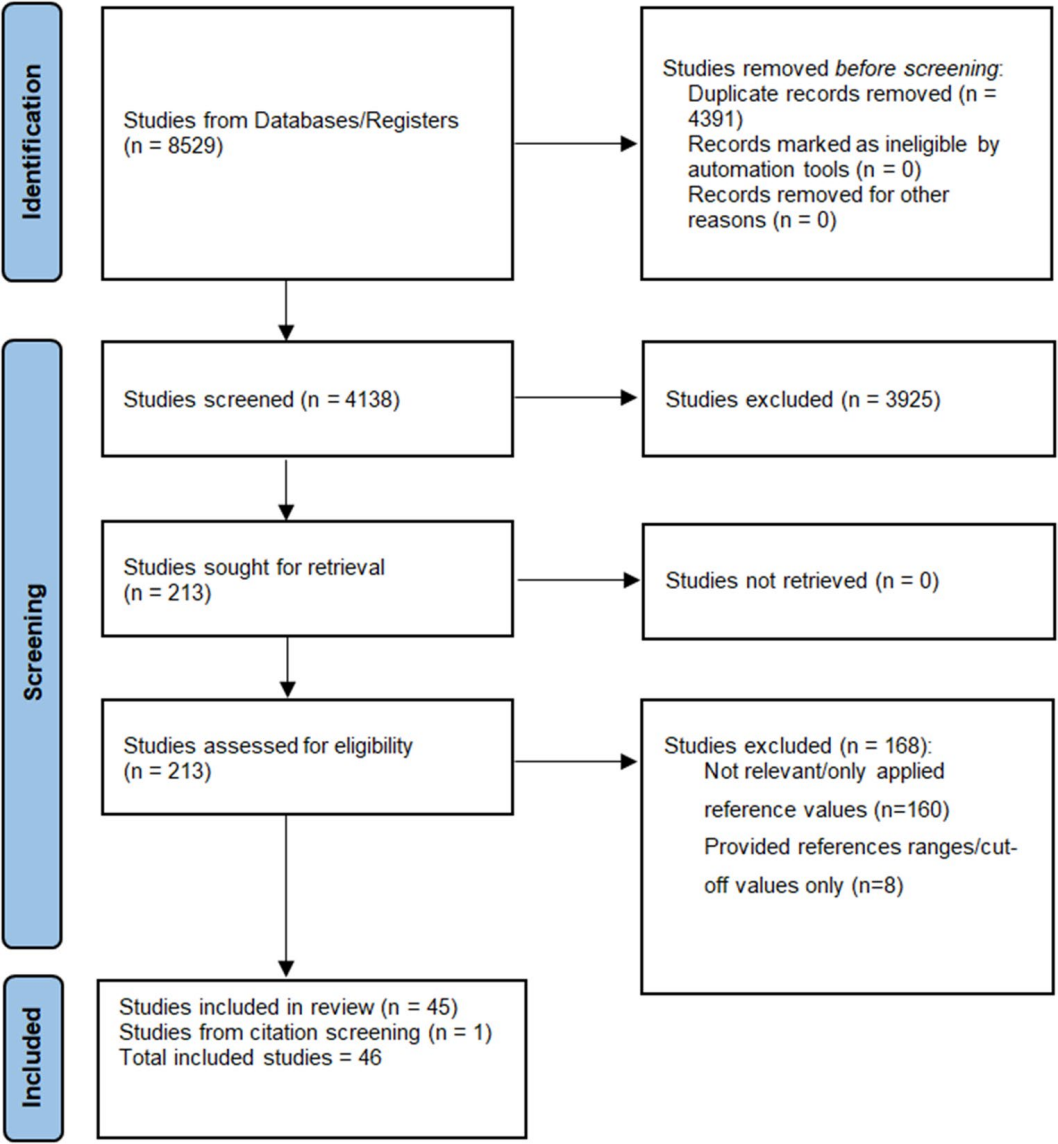
Prisma diagram of systematic literature search

A flowchart summarizing the study selection procedure is presented in Fig. 1. The screening process based on searching relevant electronic databases and additional manual search resulted in the retrieval of 8,529 articles. After removing duplicates, a total of 4,391 articles were screened. Following the initial screening based on article titles, 4,199 articles underwent abstract screening, and 213 articles were assessed based on full text, which resulted in inclusion of 46 studies. One study was identified through citation screening. Consequently, a total of 46 studies fulfilled the inclusion criteria and were included in this systematic review.

## Overview of Included Studies

**Fig. 2:**
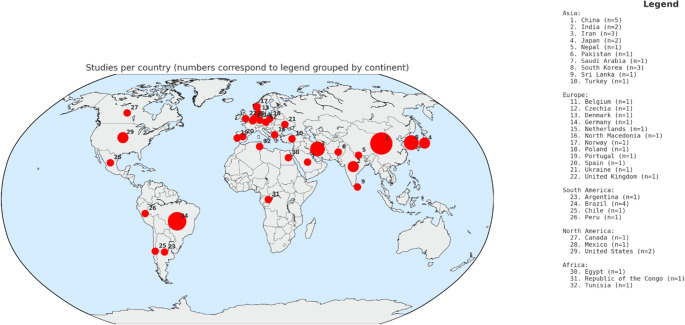
Geographical distribution of studies included in the systematic review

The characteristics of the selected studies according to MetS components are reported in Table 1. An overview of the geographical distribution of studies are shown in Fig. 2. Highest number of studies were conducted in Asia (*n* = 20; 43.5%) followed by Europe (*n* = 12; 26.1%), South America (*n* = 7; 15%), North America 4 (8.7%), and Africa (*n* = 3; 6.5%). In total, 26 (56.5%) studies were conducted in low- and middle-income countries, whereas 20 (43.5%) studies were conducted in high-income countries. Age of the participants ranged from 0 to 19 years in the included studies. Out of the 46 included studies, 41 (89.1%) included adolescents 10–14 years, whereas only 5 (10.9%) studies included children under 2 years and 18 (39.1%) studies included children aged 2–4 years (Figure S1). Ratio of girls to boys in the included studies ranged from 0.88 to 1.5. Majority (*n* = 39) of the studies had a cross-sectional design, whereas 6 studies were prospective cohort and 1 study was controlled intervention study. Sample sizes of the included studies ranged from 1,035 [18] to 68,261 [19].

**Table 1:** General characteristics of included studies according to metabolic syndrome indicators

Study No	Author, year	Country	Population	Age range (years)	Study type	Data collection (years)	Method	
	Waist Circumference							
1	Kulaga et al., 2023	Poland	22,370 (girls:11,611/boys:10,759)	3–18	Cross-sectional	2007–2012	LMS	
2	Lee et al., 2022	Korea	22,495 (girls:10,882/boys:11,613)	2–18	Cross-sectional	2007–2019	LMS	
3	Hasegawa et al., 2021	Japan	9,695 (girls:4,758/boys:4,937)	0–6	Cross-sectional	1978–1981	LMS	
4	Jáuregui-Ulloa et al., 2021	Mexico	12,979 (girls:6,998/boys:5,981)	5–17	Cross-sectional	2018	Quantile regression	
5	Marrodan Serrano et al., 2021	Argentina, Cuba, Spain -Mexico -Venezuela	13,289 (girls:6,714/boys:6,575)	6–18	Cross-sectional	2005–2011	LMS	
6	Sarna et al., 2021	India	68,261 (girls:32,814/boys:35,449)	5–19	Cross-sectional	2016–2018	LMS	
7	Van Eyck et al., 2021	Belgian	2058*	3–18	Prospective cohort	2012–2020	GAMLSS	
8	Vendula et al., 2021	Czech Republic	2,093 (girls:1,085/boys:1,008)	6–11	Cross-sectional	ND	GAMLSS	
9	Asif et al., 2020	Pakistan	10,668 (girls:5,129/boys:5,539) *	2–18	Cross-sectional	2016	LMS	
10	Bojanic et al., 2020	North Macedonia	2,490 (girls:1,202/boys:1,288)	11–18	Cross-sectional	2017	LMS	
11	Cossio-Bolaños et al., 2020	Peru	1,536 (girls:788/boys:748)	5- 17.9	Cross-sectional	2016	LMS	
12	Ghouili et al., 2020	Tunisia	2,308 (girls:1,186/boys:1,122)	6–18	Cross-sectional	2014–2015	LMS	
13	Shah et al., 2020	UK	1,562 (girls:910/boys:652)	4–13.9	Cross-sectional	2004–2007	LMS	
14	Gomez-Campos et al., 2019	Chile	9,232 (girls:4,381/boys:4,851)	6–18.9.9	Cross-sectional	2014–2015	LMS	
15	Sousa et al., 2019	Portugal	6,987(girls:3,532/boys:3,455)	6–18	Cross-sectional	2004–2009	LMS	
16	Andaki et al., 2018	Brazil	1,397(girls:729/boys:668)	6–10	Cross-sectional	2011–2012	LMS	
17	Fredriksen et al., 2018	Norway	2,271(girls:1,121/boys:1,150)	6–12	Controlled intervention	2015	ND	
18	Karki et al., 2018	Nepal	1,135*	5, 6, 12 and 15	Cross-sectional	2016	LMS	
19	Thangjam et al., 2018	India	2,334 (girls:1,093/boys:1,241)	5–15	Prospective cohort	2012–2015	ND	
20	Zong et al., 2018	China	53,172 (girls:26,521/Boys:26,651)	3–7	Cross-sectional	2015	ND	
21	Gromnatska et al., 2024	Ukraine	1,566 (girls: 807/boys: 759)	10–17	Cross-sectional	ND	ND	
22	Alves Junior et al., 2024	Brazil	9,665 (girls: 5023/boys: 4642)	7–14	Cross-sectional	2002–2019	LMS	
	**Systolic and diastolic blood pressure**							
1	Fujita et al., 2023	Japan	3,361(girls:1,689/boys:1,672)	2	Cross-sectional	2015–2017	LMS	
2	Ramgopal et al., 2023	United States	343,129*	0–17	Cross-sectional	2020–2021	GAMLSS	
3	Ahmadi et al., 2020	Iran	1,035(girls:579/boys:456)	6–18	Cross-sectional	2017	LMS	
4	AlSalloum et al., 2020	Saudi Arabia	2,553(girls:1,254/boys:1,299)	2–6	Cross-sectional	2004–2005	Mixed-effect linear regression	
5	Jardim et al., 2020	Brazil	73,999*	12–17	Cross-sectional	2009	Polynomial-regression	
6	Keskinoglu et al., 2020	Turkey	4,984 (girls:2,486/boys:2,498)	2–17	Cross-sectional	2012–2013	Polynomial-regression	
7	Lee et al., 2020	Korea	1,732 (girls:868/boys:864)	3, 5,7,8,9	Cross-sectional	2001–2006	GAMLSS	
8	Kim et al., 2019	Korea	10,442 (girls:4,953/boys:5,489)	10–18	Cross-sectional	1998–2016	GAMLSS	
9	El-Shafie et al., 2018	Egypt	60,025 (girls:28,422/boys:31,603)	0–19	Cross-sectional	2015–2017	Regression/ND	
10	Muyumba et al., 2018	Republic Kongo	6,883 (girls:3,510/boys:3,373)	3–17	Cross-sectional	2014–2016	GAMLSS	
11	Sooriyakanthan et al., 2018	Sri Lanka	1,922 (girls:972/boys:950)	6–18	Cross-sectional	ND	Linear regression	
	**Biomarkers of glucose metabolism and insulin resistance**							
1	Hovestadt et al., 2022	Germany	2,455 (girls:1,190/boys:1,265)	0.5–18	Prospective cohort	2011–2017	GAMLSS	
2	Hu et al., 2021	United States	7786 (girls:3946/boys:3840)	12–20	Cross-sectional	1999–2018	Quantile regression	
3	Chissini et al., 2020	Brazil	37,815 (girls:22,682/boys:15,133)	12–17	Cross-sectional	2013–2014	ND	
4	Ata et el, 2018	Canada	6,116 (girls:2,963/boys:3,153)	6–19	Cross-sectional	2007–2013	LMS	
5	Alías-Hernández et al., 2018	Spain	654 (girls:336/boys:318)	2–9.9.9	Cross-sectional	2009	GAMLSS	
6	Ren et al., 2024	China	4,615 (girls:2293/boys:2322)	3–12	Cross-sectional	2018–2019	GAMLSS	
	**Biomarkers for lipid metabolism**							
1	Montazeri- Najafabady et al., 2023	Iran	472 (girls:234/boys:238)	9–18	Prospective cohort	ND	LMS	
2	Li et al., 2021	China	15,830 (girls:7,757/boys:8,073)	6–17	Cross-sectional	2013	LMS	
3	Azizi-Soleiman et al., 2020	Iran	3,843 (girls:1,833/boys:2,010)	7–18	Cross-sectional	2015	ND	
4	Xiao et al., 2019	China	12,875 (girls:6,250/boys:6,625)	6–18	Cross-sectional	2013–2015	GAMLSS	
5	Ata et el, 2018	Canada	6,116 (girls:2,963/boys:3,153)	6–19	Cross-sectional	2007–2013	LMS	
6	Balder et al., 2018	Netherlands	8,071 (girls:4248/boys:3,823)	8–18	Cross-sectional	2006–2013	GAMLSS	
7	Greve et al., 2024	Denmark	1,456 (girls:751/boys: 705)	5–17	Prospective cohort	2008–2015	GAMLSS	
8	Yu et al., 2024	China	5,624 (girls. 2715/boys: 2909)	0–15	Prospective cohort	2017–2022	LMS	

* sex specific numbers not provided

Overall, 22 studies [11, 19–39] reported reference values for WC, 11 studies [12, 13, 18, 40–47] for systolic and diastolic blood pressure, 6 studies [48–53] for HbA1c [48, 51, 53], fasting glucose [49], insulin [51, 52] and HOMA-IR [50–52]) and 8 studies [51, 54–60] for lipid parameters (TC, LDL, HDL, and TG). Majority of the studies focused on single metabolic indicators except Ata N. et al. [51] that reported reference curves for HbA1c, HOMA-IR, TC and HDL-C.

The assessment methods for WC, blood pressure, and individual metabolic biomarkers are summarised in Table 2. WCwas measured consistently across studies, most commonly at the midpoint between the lowest rib and the iliac crest using a non-stretchable tape. In contrast, variations were observed in the methods for blood pressure measurement and biomarker assessments.

**Table 2:** Measurements used for evaluation of biomarkers/anthropometric indicators

SNo	Author, year	Metabolic indicator indicators evaluated	Measurement method		
	Waist Circumference				
1.	Kulaga et al., 2023	WC	Measured midway between the lowest rib and the iliac crest without clothes at the end of expiration using non-stretch anthropometric tape		
2.	Lee et al., 2022	WC	Measured midway between the lowest rib and the iliac crest (highest margin) at the end of expiration using flexible tape (Seca 220)		
3.	Hasegawa et al., 2021	WC	Measured at the level of the umbilicus. 0–1 year: Supine position 2–7 years: Standing position		
4.	Jáuregui-Ulloa et al., 2021	WC	Measured midway between the lower costal border and the iliac crest at the end of expiration using rigid metal tape (Lufkin W606PM, Lufkin, NC, USA)		
5.	Marrodan Serrano et al., 2021	WC	Measured at the umbilical level		
6.	Sarna et al., 2021	WC	Measured midway between the lowest rib and the iliac crest (highest margin) at the end of expiration using flexible tape		
7.	Van Eyck et al., 2021	WC	Measured approximately 1 cm above the umbilicus at the point of the smallest circumference between the lowest rib and highest hip comb. Standing position		
8.	Vendula et al., 2021	WC	Measured at the point just above the uppermost lateral border of the right iliac crest, at the end of a normal exhalation		
9.	Asif et al., 2020	WC	Measured midway between the lowest rib and the iliac crest (highest point) at the end of expiration using non-elastic plastic tape		
10.	Bojanic et al., 2020	WC	Measured midway between the lowest rib and the iliac crest (highest point) at the end of expiration using non-elastic anthropometric tape		
11.	Cossio-Bolaños et al., 2020	WC	Process not described, used metal Seca tape		
12.	Ghouili et al., 2020	WC	Measured midway between the lowest rib and the iliac crest (highest margin) using non-elastic flexible tape		
13.	Shah et al., 2020	WC	Measured approximately midway between the lowest rib and the iliac crest (highest margin) using non-elastic retractable tape and adjusted for clothing (0.5 cm)		
14.	Gomez-Campos et al., 2019	WC	Measured midway between the lowest rib and the iliac crest (highest margin) using metal anthropometric measuring tape (Seca brand)		
15.	Sousa et al., 2019	WC	Measured midway between the lowest rib and the iliac crest at the end of expiration using non-extendable tape		
16.	Andaki et al., 2018	WC	Measured midway between the lowest rib and the iliac crest at the end of expiration using a flexible and non-elastic tape (Sanny, São Paulo, Brazil)		
17.	Fredriksen et al., 2018	WC1, WC2	Measured at the umbilicus level after normal expiration using non-elastic measuring tape		
18.	Karki et al., 2018	WC	Measured midway between the lowest rib and the iliac crest using inelastic plastic measuring tape (Prym^®^, William Prym Holding GmbH, Stolberg, Germany)		
19.	Thangjam et al., 2018	WC	Measured midway between the lowest rib and the iliac crest using non-stretchable tape during end-tidal expiration		
20.	Zong et al., 2018	WC	Measured midway between the lowest rib and the iliac crest at the end of expiration using inextensible plastic tape Standing position		
21.	Gromnatska et al., 2024	WC	Measured midway between the lower rib and the ilium crest using flexible measuring tape		
22.	Alves Junior et al., 2024	WC	Measured midway between the lower rib and the ilium crest using fibre anthropometric tape (Sanny^®^, model TR4013, São Paulo, Brazil)		
	**Systolic and diastolic blood pressure**				
1.	Fujita et al., 2023	SBP and DBP	Aneroid sphygmomanometer (DS66 DuraShockTM hand aneroid [Welch Allyn Inc, Syracuse, NY, USA] Sitting position Three measurements but the average value of two consecutive measurements		
2.	Ramgopal et al., 2023	SBP and DBP	Automated blood pressure monitors From Arm or Leg		
3.	Ahmadi et al., 2020	SBP and DBP	Automatic digital BP device (Automatic Blood pressure Monitor, Model M3 Comfort, Omron Co., Osaka, Japan). Sitting position, right hand Three measurements taken, average of last two used		
4.	AlSalloum et al., 2020	SBP and DBP	Oscillometric techniques readings Children < 2 years: supine position Children > 2 years in sitting position. Recorded the Lower BP level of two measurements		
5.	Jardim et al., 2020	SBP and DBP	Oscillometric device Three BP measurements (The mean from the two last measurements were used)		
6.	Keskinoglu et al., 2020	SBP and DBP	Oscillometric device Right arm, heart level Three readings of BP, with a 2-minute interval Average of the three measurements		
7.	Lee et al., 2020	SBP and DBP	Automated instrument (Dinamap Procare 200; GE Inc., Milwaukee, WI, USA) Right arm, heart level Two BP measurements, with a 5-minute interval Average of the two measurements		
8.	Kim et al., 2019	BP, SBP, DBP	Mercury sphygmomanometer (Baumanometer sphygmomanometer, W.A. Baum Co Inc., Copiague, NY, USA) & Littmann Stethoscope (3 M, Maplewood, MN, USA) Right arm, heart level Mean of the second and third readings		
9.	El-Shafie et al., 2018	SBP and DBP	Standard mercury sphygmomanometers (Model 1002/Presameter, Riester, Germany) Infants: supine position Children: in sitting position Two readings, with 5–10 min interval The mean of the readings		
10.	Muyumba et al., 2018	SBP and DBP	Oscillometric measurement (Datascope Accutorr Plus; Datascope Corporation, USA) Sitting position, heart level 30 min after physical exercise or last meal Three readings, 1-minute Interval Mean of the second and third reading		
11.	Sooriyakanthan et al., 2018	SBP and DBP	Standard mercury sphygmomanometer Three readings with 5-minute interval. Average of second and third readings was used		
	**Biomarkers of glucose metabolism and insulin resistance**				
1.	Hovestadt et al., 2022	HbA1c	COBAS 8000 c502 platform Turbidimetric immunological inhibition assay (TINIA) using hemolyzed blood Results given in NGSP units [%]		
2.	Hu et al., 2021	blood glucose	ND		
3.	Chissini et al., 2020	HOMA-IR	Blood samples after 12-h overnight fast Hexokinase method for plasma glucose using ADVIA 2400 Clinical Chemistry System Electrochemiluminescence assays for Insulin Modular E170s (Roche, Indianapolis, IN, USA) and Enzymatic colorimetric method for lipid profil Modular Analyser (Roche, Indianapolis, IN, USA) HOMA-IR with equation proposed by Matthews et al.		
4.	Ata et el, 2018	HbA1c, Insulin, HOMA-IR	Insulin: Solid-phase, chemiluminescent immunometric assay Analyzer: Advia Centaur XP (Siemens) HOMA-IR: Calculated as (fasting insulin [µU/L] x glucose [mmol/L])/22.5 HbA1c: Immunoturbidimetric test, Analyzer: Vitros 5,1FS (Ortho Clinical Diagnostics)		
5.	Alías-Hernández et al., 2018	insulin, HOMA-IR	Blood Samples: overnight fasting > 8 h; Glucose & Lipid Profil: Cobas e-501 analyser (Roche Diagnostics, Basel, Switzerland) Insulin: Cobas c-601 analyser (Roche Diagnostics, Basel, Switzerland) HOMA index: (glucose [mmol/L] × insulin [µIU/mL])/22.5 QUICKI: (1/log [fasting insulin] + log [fasting glucose])		
6.	Ren et al., 2024	HbA1c	A1C EZ 2.0 POC analyzer (BioHermes Ltd., Wuxi, Jiangsu, China).		
	**Triglycerides and HDL cholesterol**				
1.	Montazeri- Najafabady et al., 2023	TC, HDL, LDL, TG	Serum total cholesterol, HDL-C, and triglycerides (TG): Enzymatic reagents (Biosystems, Barcelona, Spain), A-25 Biosystem Autoanalyser LDL: Friedwald equation from calculated TG, HDL-C, and total cholesterol (TC) Non-HDL-C: subtracting HDL-C from total cholesterol		
2.	Li et al., 2021	TC, LDL-C, HDL-C, TG	TC, LDL-C, HDL-C and TG: enzymatic methods LDL-C and HDL-C: clearance methods		
3.	Azizi-Soleiman et al., 2020	TG, TC, LDL-C, HDL-C, (1, 2)	Lipid profiles (TG, TC, LDL-C, HDL-C): Enzymatic colorimetric method Analyzer: Hitachi Automatic Analyzer 7600, LDL-C direct measurement		
4.	Xiao et al., 2019	TC, LDL-C, HDL-C, TG	Total cholesterol (TC), LDL-C, HDL-C, triglycerides (TG): Enzymatic method Analyzer: Hitachi 7080 automated analyzer		
5.	Ata et el, 2018	TC, HDL	Colorimetric test Vitros 5,1FS analyzer (Ortho Clinical Diagnostics)		
6.	Balder et al., 2018	TC, HDL, LDL, TG	Total cholesterol (TC), LDL-C, HDL-C, triglycerides (TG): Vitros 5,1FS (Ortho Clinical Diagnostics)		
7.	Greve et al., 2024	TC, TG, LDL, HDL, remnant cholosterol	Fasting blood samples, enzymatic colorimetric method on a Roche/Hitachi cobas c system machine		
8.	Yu et al., 2024	TG, TC, HDL-C, LDL-C	TG, TC, HDL-C: Roche cobas702 automatic biochemical analyzer, enzymatically measured		

Abbreviations: DBP: Diastolic blood pressure; HbA1c: Hemoglobin A1c; HDL-C: High Density Lipoprotein Cholesterol; HOMA-IR: homeostasis model assessment of insulin resistance; HTN: hypertension; LDL-C: Low Density Lipoprotein Cholesterol; ND: Not described; SBP: Systolic blood pressure; TC: Total cholesterol; TG: Triglycerides; WC: Waist circumference

### Statistical Modelling Approaches for Deriving Reference Curves

The reference curves in the different studies were derived predominantly by generating smoothed reference centile curves based on the Lambda-Mu-Sigma (LMS) method (*n* = 22) and the Generalized Additive Models for Location, Scale, and Shape GAMLSS method (*n* = 12). LMS is an approach to construct the normalized percentiles using Box-Cox transformation that summarizes the distribution through the median, coefficient of variation, and skewness [61]; whereas GAMLSS is a generalized regression approach to construct normalized percentiles using not only location, scale, and skewness but also kurtosis, allowing greater flexibility in modelling age-dependent distributional changes [62]. Other approaches used for percentile estimation included polynomial regression (*n* = 2), quantile regression (*n* = 2) and linear regression (*n* = 2). Six studies [34, 36–38, 50, 56] did not specify or provide details of the statistical method used to estimate percentiles for the reference curves.

### Percentile Distribution According To Metabolic Indicators

#### Waist Circumference

**Fig. 3:**
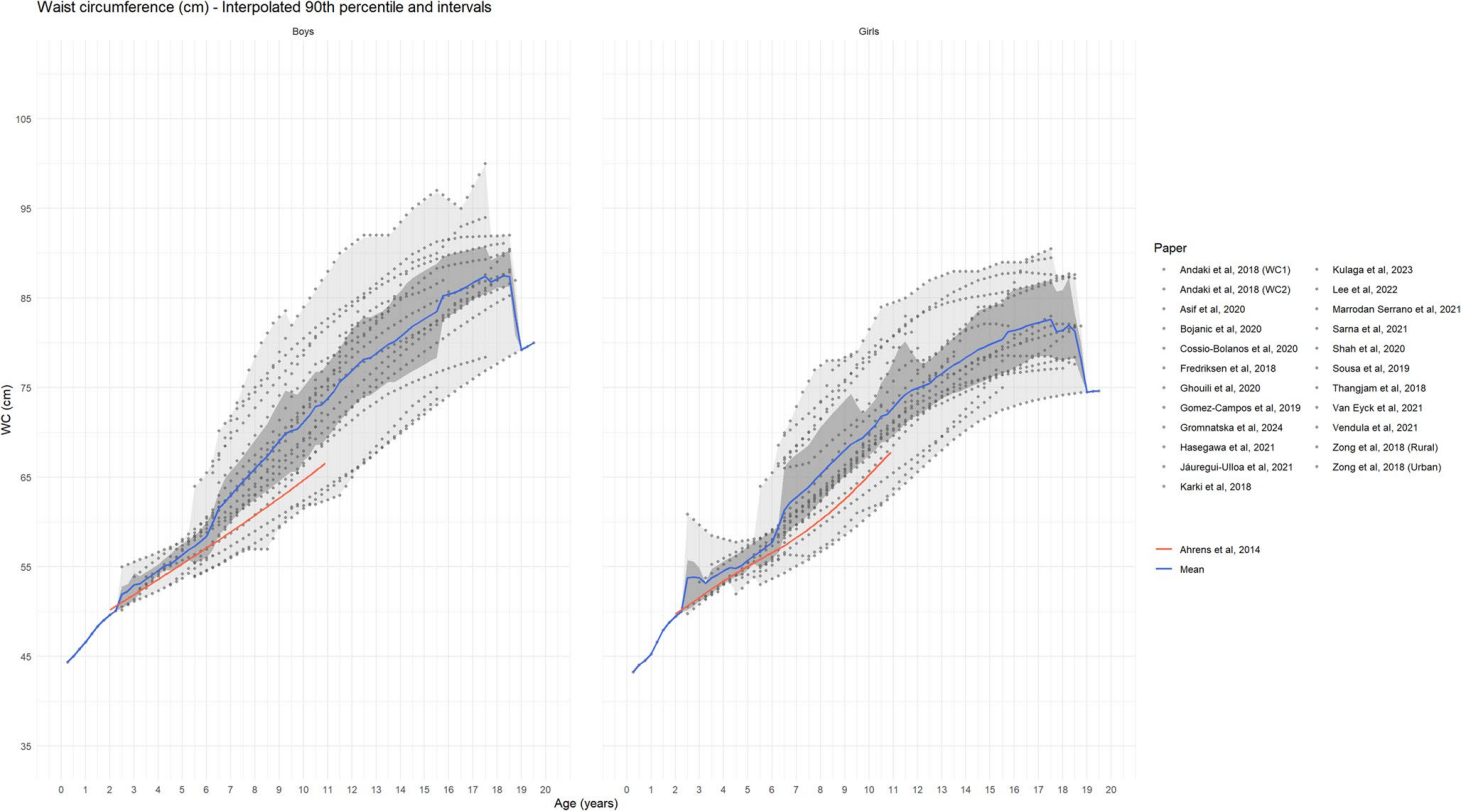
Reference values for WC by age and sex (absolute values) 90th percentile curves of included studies, corresponding intervals bands covering all studies (light grey) and 50% of studies (dark grey) and mean 90th percentile The red line shows 90th percentile reference from IDEFICS definition by Ahrens et al., 2014 [5]

WC reference values were derived for the healthy paediatric populations in 22 studies. The respective age- and sex-specific percentile curves reported by the individual studies are presented in Table 3 and the corresponding 90th percentiles are shown in Fig. 3. Due to differences in age-groups across studies, the reported percentiles did not cover the whole age range from 2 to 19 years in each study. Overall, the 50th and 90th percentile of the WC showed a steady increase in WC with age in both sexes (Figure S2). These results are consistent with the reference values previously reported based on the IDEFICS study population of European children [5]. Comparison of the study-specific 50th and 90th percentiles showed larger inter-study variations in WC for corresponding ages. The mean 50th percentile of the included studies agreed well with the IDECFICS reference values; however, the mean 90th percentile of the included studies was higher than the IDEFICS 90th percentile, with larger difference observed in higher age-groups. In these studies, sex-specific 90th percentile [19, 22–25, 27–29, 32, 38] was the most common cut-off to define abdominal obesity in children and adolescents. Some studies suggested 95th [27, 29] or 75th [37] percentiles as cut-off for higher risk/obesity among children and adolescents (Table 3).

**Table 3:** Percentile distribution according to metabolic syndrome components

Author, Year	Biomarkers/Anthropometric indicators evaluated	Percentiles reported	Stratification variables and modeling of reference growth curves	Diagnostic
Waist circumference				
Kulaga et al., 2023	WC	P3, P10, P25, P50, P75, P90, P95 shown in percentile curves; values reported for P90, P95	Sex specific, Age specific	Abdominal obesity: WC: P90 and P95 and cut-offs linked to adult cutoffs: boys 94 cm, girls: 80 cm
Lee et al., 2022	WC	P3, P5, P10, P25, P50, P75, P90, P95, P97	Sex specific, Age specific	Abdominal obesity: WC ≥ P90
Hasegawa et al., 2021	WC	P3, P10, P25, P50, P75, P90, P97	Sex specific, Age specific	P90 both sexes for normal weight Abdominal obesity: P97 boys; P96 girls
Jáuregui-Ulloa et al., 2021	WC	P5, P10, P15, P20, P25, P30, P40, P50, P60, P70, P75, P80, P85, P90, P95	Sex specific, Age specific	Abdominal obesity: WC ≥ P90
Marrodan Serrano et al., 2021	WC	P3, P5, P10, P25, P50, P75, P90, P95, P97	Sex specific, Age specific	Abdominal obesity: WC ≥ P90
Sarna et al., 2021	WC	P5, P25, P50, P75, P85, P90, P95	Sex specific, Age specific	Abdominal obesity: WC ≥ P90
Van Eyck et al., 2021	WC	P3, P5, P10, P25, P50, P75, P90, P95, P97	sex specefic, Age specific	ND
Vendula et al., 2021	WC	P3, P10, P25, P50, P75, P90, P97	Sex specific, Age specific	ND
Asif et al., 2020	WC	P5, P10, P25, P50, P75, P90, P95	Sex specific, Age specific	Lower: P5, Higher Abdominal obesity: P95, Critical Cut-off (Abdominal Obesity): P90
Bojanic et al., 2020	WC	P3, P10, P25, P50, P75, P90, P97	Sex specific, Age specific	Midpoint (mean): P50, Abdominal obesity: P90
Cossio-Bolaños et al., 2020	WC	P3, P5, P10, P15, P25, P50, P75, P85, P90, P95, P97	Sex specific, Age specific	Underweight: P5, Abdominal obesity: P95
Ghouili et al., 2020	WC	P3, P10, P25, P50, P75, P90, P97	Sex specific, Age specific	Optimal Percentiles of WC for cardiovascular disease P75
Shah et al., 2020	WC	P2, P9, P25, P50, P75, P90, P91, P98, P99,6	Sex specific, Age specific	Abdominal Obesity WC ≥ P90
Gomez-Campos et al., 2019	WC	P3, P5, P10, P15, P50, P85, P95, P97	Sex specific, Age specific	ND
Sousa et al., 2019	WC	P3, P5, P10, P25, P50, P75, P85, P90, P95, P97	Sex specific, Age specific	ND
Andaki et al., 2018	WC	P5, P10, P25, P50, P75, P90, P95	Sex specific, Age specific	ND
Fredriksen et al., 2018	WC	P5, P10, P25, P50, P75, P90, P95	Sex specific, Age specific	ND
Karki et al., 2018	WC	P3, P10, P25, P50, P75, P90, P97	Sex specific, Age specific	Abdominal Obesity: WC cut-offs of + 1.28 SDS or > 90th percentile
Thangjam et al., 2018	WC	P5, P10, P25, P50, P70, P75, P90, P95	Sex specific, Age specific	ND
Zong et al., 2018	WC	P5, P10, P15, P20, P25, P50, P75, P80, P85, P90, P95	Sex specific, Age specific	Increased risk of cardiovascular factors P75 & P90 (in China)
Gromnatska et al., 2024	WC	P5, P10, P25, P50, P75, P90, P95	Sex specific, Age specific	Abdominal Obesity: WC ≥ P90
Alves Junior et al., 2024	WC	P5, P10, P25, P50, P75, P85, P95	Sex specific, Age specific	ND
**Systolic and diastolic blood pressure**				
Fujita et al., 2023	SBP and DBP	P50, P90, P95, P99 for following percentiles of height: P5, P10, P25, P50, P75, P90, P95	Sex specific, Height specific	ND
Ramgopal et al., 2023	DBP	P1, P2.5, P5, P10, P25, P50, P75, P90, P95, P97.5, P99	Age specific	Abnormal DBP (a DBP < 10th or > 90th centile)
Ahmadi et al., 2020	SBP and DBP	P50, P90, P95, P99 for following percentiles of height: P5, P10, P25, P50, P75, P90, P95	Sex specific, Age specific, Height specific	Pre-hypertension: SBP & DBP > P90, Stage I Hypertension: SBP & DBP > P95 + 5mmHg, Stage II hypertension SBP & DBP > P99boysgirls + 5mmHg
AlSalloum et al., 2020	SBP and DBP	P50, P90, P95, P99, P95 + 12 mm Hg for following percentiles of height: P5, P10, P25, P50, P75, P90, P95	Sex specific, Age specific, H eight specific	Normal BP < P90, Elevated BP ≥ P90, Stage I Hypertension BP ≥ P95 to BP < P95 + 12mmHg, Stage II hypertension BP ≥ P95 + 12mmHg or ≥ 140/90mmHgboysgirls
Jardim et al., 2020	SBP and DBP	P50, P90, P95, P99 for following percentiles of height: P5, P10, P25, P50, P75, P90, P95	Sex specific, Age specific, Height specific	No clear definition of hypertension
Keskinoglu et al., 2020	SBP and DBP	P50, P90, P95 for following percentiles of height: P5, P25, P50, P75, P95	Sex specific, Age specific,Height specific	Normal BP < P90 percentile Preadolescent, prehypertension SBP and DBP ≥ P90 and < P95 Hypertension: SBP and DBP ≥ P95boysgirls
Lee et al., 2020	SBP and DBP	P5; P10; P25; P50; P75; P90; P95 for following percentiles of height: P50, P90, P95	Sex specific, Age specific, H eight specific	Prehypertension between P90 and P94 hypertension: SBD, DPB: ≥ P90
Kim et al., 2019	SBP and DBP	P50; P90; P95; P99 for following percentiles of height: P5, P10, P25, P50, P75, P90, P95	Sex specific, Age specific, Height specific	Hypertension: SBD, DPB: ≥ P95
El-Shafie et al., 2018	BP, SBP, DBP	P50, P75, P90, P95	Sex specific,Age specific	Normal SB: P50-P90boysgirls, high-normal SB: P90-P95boysgirls, high BP > P95
Muyumba et al., 2018	SBP and DBP	P50; P90; P95 for following percentiles of height: P5, P25, P50, P75, P95	Sex specific, Age specific, Height specific	Prehypertension SBP & DBP ≥ P90 Hypertension SBP & DBP ≥ P95 define Metabolic Syndrome: BP ≥ P90
Sooriyakanthan et al., 2018	SBP and DBP	P50, P90, P95 for following percentiles of height: P25; P50; P75	Sex specific, age specific, Height specific	ND
**Biomarkers of glucose metabolism and insulin resistance**				
Hovestadt et al., 2022	HbA1c	P2,5, P5, P10, P25, P50, P75, P95, P97,5	Sex specific, Age specific	ND
Hu et al., 2021	blood glucose	P25, P50, P75	Sex specific, Age specific	ND
Chissini et al., 2020	HOMA-IR	P5, P25, P50, P75, P90	Sex specific, Age specific	Higher Blood Pressure: P90, MetS Cut-off: P75 girls; P90 boys
Ata et el, 2018	HbA1c, Insulin, HOMA-IR	P3, P10, P25, P50, P75, P90, P97	Sex specific, Age specific	ND
Alías-Hernández et al., 2018	insulin, HOMA-IR	P25, P50, P75, P90	Sex specific, Age specific	Insulinaemia > P90, HOMA-IR > P90
Ren et al., 2024	HbA1c	P1, P3, P5, P10 P25, P50, P75, P90, P95, P97, P99	Sex specific, Age specific	ND
**Biomarkers for lipid metabolism**				
Montazeri- Najafabady et al., 2023	TC, HDL, LDL, TG	P3, P10, P25, P50, P75, P90, P97	Sex specific, Age specific	TC ≥ 200 mg/dL, LDL-C ≥ 130 mg/dL, HDL C < 40 mg/dL. Furthermore, the recommended thresholds for defining hyper triglyceridemic have been ≥ 100 mg/dL and ≥ 130 mg/dL in children aged 0–9 and 10–19 years, respectively [31]. In the present study, the 97th percentile for TG,
Li et al., 2021	TC, LDL-C, HDL-C, TG	P2,5, P5, P10, P25, P50, P75, P90, P95, P97,5	Sex specific, Age specific	Familial hypercholesterolemia: - HDL: (P2,5) LDL-C, TC, TG: (P97,5) LDL-C, TC, TG: ≥ P95 high HDL-C < P10 low
Azizi-Soleiman et al., 2020	TG, TC, LDL-C, HDL-C	P5, P10, P25, P50, P75, P90, P95	Sex specific, Age specific	pediatric dyslipidaemia: TC ≥ 200 mg/dL, LDL-C ≥ 130 mg/dL, HDL-C < 40 mg/dL, hypertriglyceridemia has been ≥ 100 mg/dL and ≥ 130 mg/dL in children aged 0–9 and 10–19 years
Xiao et al., 2019	TC, LDL-C, HDL-C, TG	TC: P5, P25, P50, P75, P95, P98, P9988 LDL-C: P5, P25, P50, P75, P95, P97, P99.5 HDL-C: P5, P12, P25, P50, P75, P95 TG: P5, P25, P50, P75, P93, P95, P98	Sex specific, Age specific	TC: - Borderline-High: boys (P98), girls (P97) - high: boys (P99.8), girls (P99.6) LDL-C: - Borderline-High: boys (P97), girls (P97) - high: boys (P99.5), girls (99.4) HDL-C: - low: boys (P12), girls (P5) TG: - Borderline-High: boys (P93), girls (P97) - high: boys (P98), girls (99.3)
Ata et el, 2018	TC, HDL	P3, P10, P25, P50, P75, P90, P97	Sex specific, Age specific	ND
Balder et al., 2018	TC, HDL, LDL, TG	P5, P10, P25, P50, P75, P90, P95	Sex specific, Age specific	ND
Greve et al., 2024	TC, TG, LDL, HDL, remnant cholosterol	P2,5, P5, P10, P25, P50, P75, P90, P95, P97,5	Sex specific, Age specific	95% Reference interval: total cholesterol boys = 2.88–5.38 mmol/l and girls = 3.00–5.79 mmol/l, HDL cholesterol boys = 0.94–2.22 mmol/l and girls = 0.92–2.33 mmol/l, LDL cholesterol boys = 1.21–3.51 mmol/l and girls = 1.32–3.76 mmol/l, triglycerides boys = 0.31–1.36 mmol/l and girls = 3.00–5.79 mmol/l and remnant cholesterol boys = 0.08–0.63 and girls = 0.11–0.70
Yu et al., 2024	TG, TC, HDL-C, LDL-C, nHDL-C	P2,5, P5, P10, P25, P50, P75, P90, P95, P97,5	Sex specific, Age specific	Dyslipidemia: TG mmol/L (mg/dL) ≥ 1.58 (140) TC mmol/L (mg/dL) ≥ 5.70 (220) HDL-C mmol/L (mg/dL) ≤ 1.04 (40) LDL-C mmol/L (mg/dL) ≥ 3.63 (140)

Abbreviations: DBP: Diastolic blood pressure; HbA1c: Hemoglobin A1c; HDL-C: High Density Lipoprotein Cholesterol; HOMA-IR: homeostasis model assessment of insulin resistance; HTN: hypertension; LDL-C: Low Density Lipoprotein Cholesterol; ND: Not described; SBP: Systolic blood pressure; TC: Total cholesterol; TG: Triglycerides; WC: Waist circumference

**Fig. 4:**
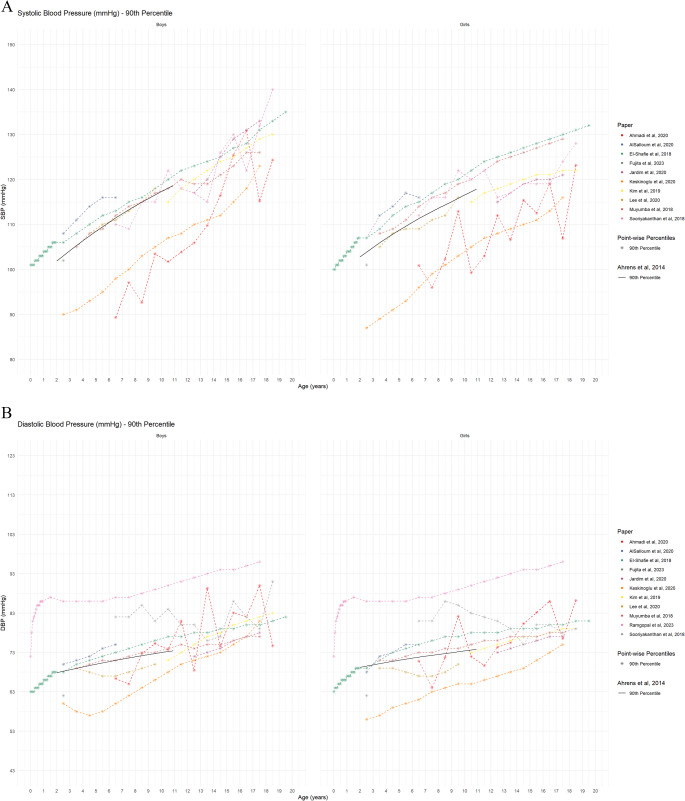
Reference values for systolic blood pressure (Panel A) and diastolic blood pressure (Panel B) by age and sex (absolute values) The black line shows 90th percentile reference from IDEFICS definition by Ahrens et al., 2014 [5]

**Fig. 5:**
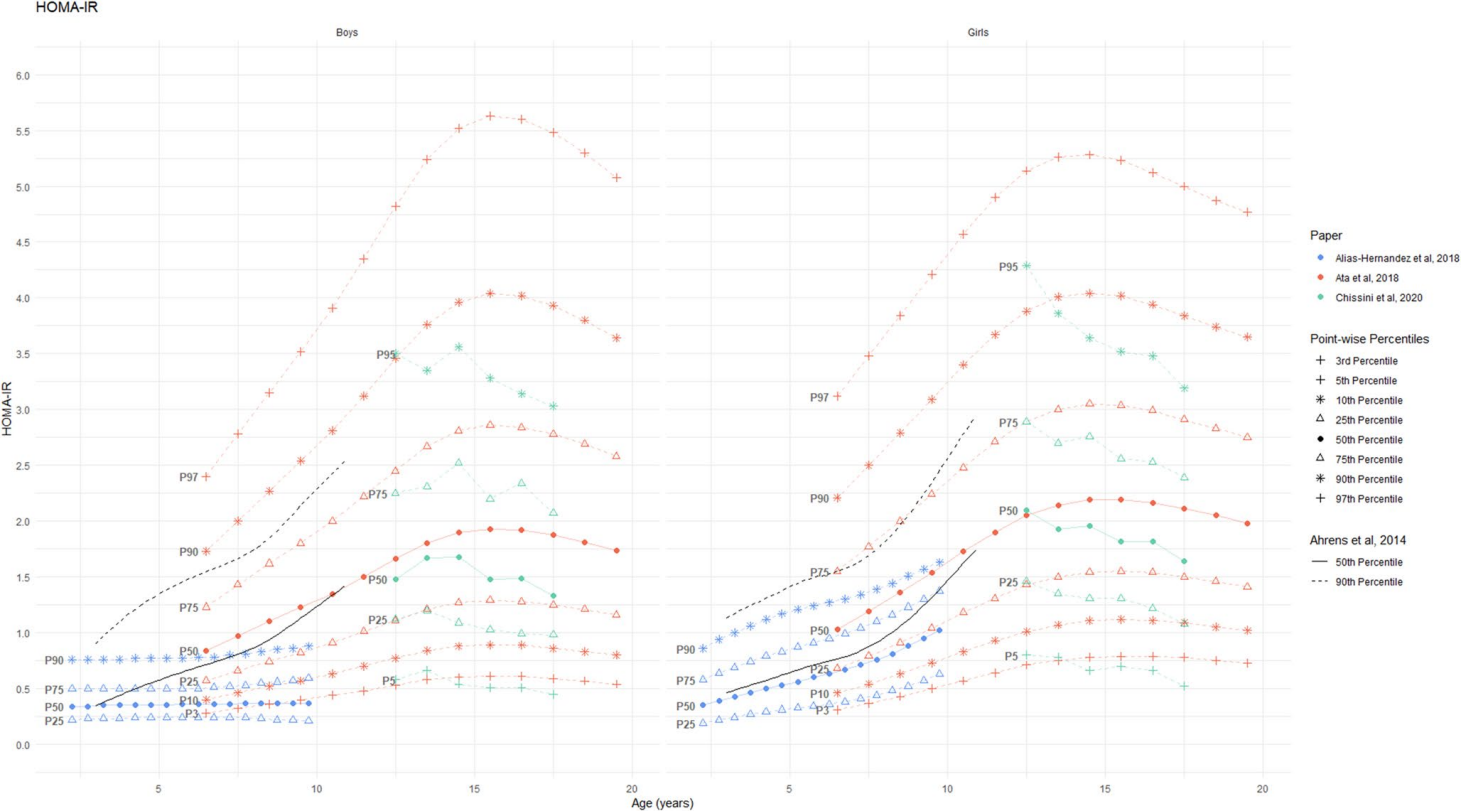
Reference values for HOMA-IR by age and sex (absolute values) The black lines show 50th and 90th percentiles from IDEFICS definition by Ahrens et al., 2014 [5]

*Blood pressure*: Overall, eleven independent studies collectively provided reference values for both SBP and DBP (Table 3). A comparative analysis of the study-specific reference values demonstrated considerable variability in the age- and sex-specific 50th (Figure S3) and 90th (Figures: 4 & 5) values across the evaluated studies. Most of the studies proposed age-, sex-, height-specific 90th [12, 13, 18, 41, 42, 46] or 95th [12, 45] percentiles of SBD and/or DBP as diagnostic cut-off to define pre-hypertension/hypertension (Figs. 4). Two studies did not propose any diagnostic criteria [40, 47] (Table 3). Although, the 90th percentiles of DBP varied across studies, the 90th percentiles of the included studies spread around the 90th percentile of IDEFICS study definition [5] (Figures: 4a & 4b).

#### Biomarkers for glucose metabolism/insulin resistance

**Fig. 6:**
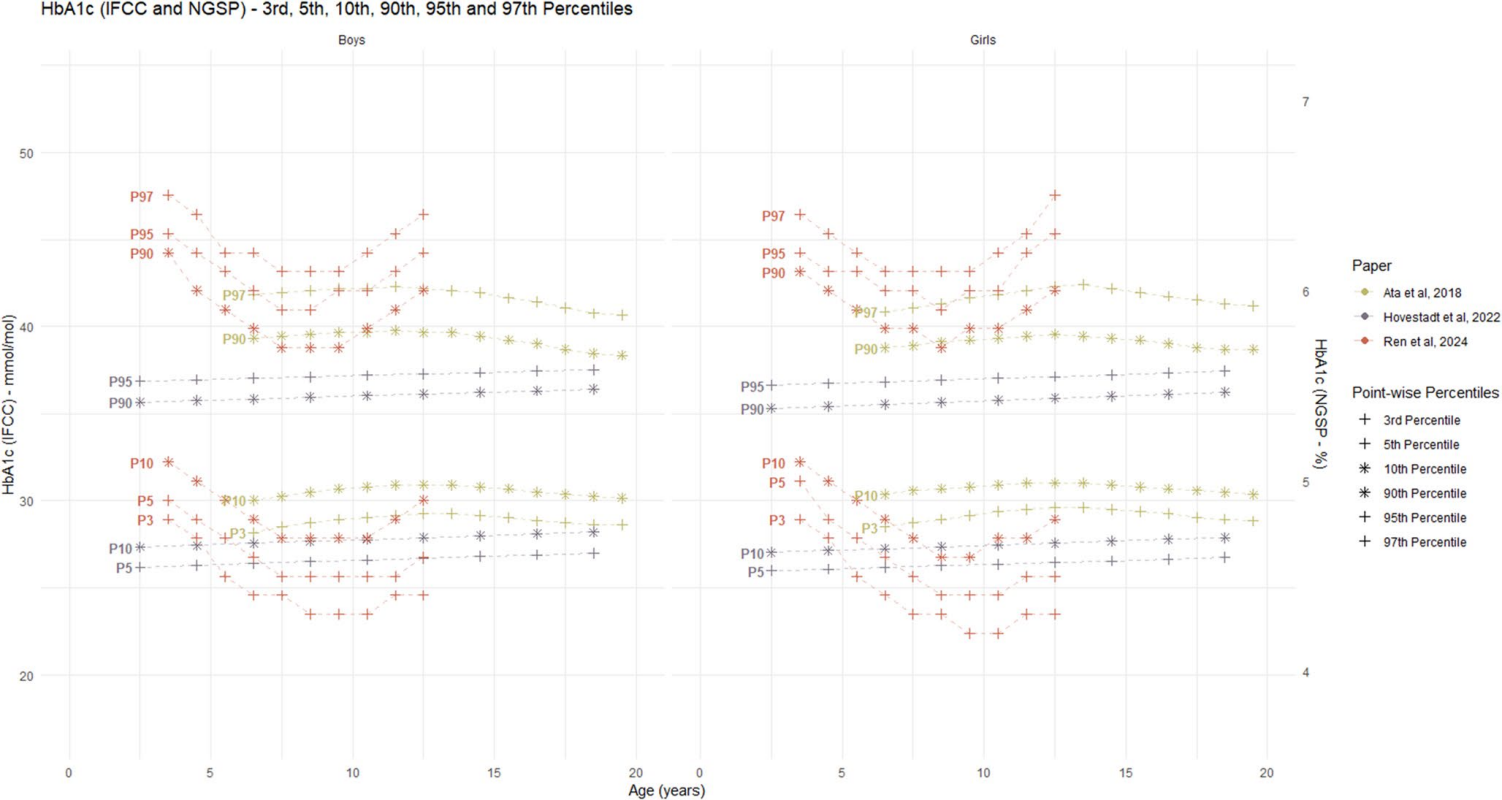
Reference values for HbA1c by age and sex (absolute values) 3rd, 5th, 10th and 90th, 95th and 97th percentile curves for HbA1c from the included studies The black lines show 50th and 90th percentiles from IDEFICS definition by Ahrens et al., 2014 [5]

Three studies developed HOMA-IR reference curves for various age groups (Table 3). Due to differences in the age-groups across different studies, these reference curves were not directly comparable. Chissini et al. [50] used 90th percentile as a threshold to identify elevated HOMA-IR values among 12–17 years-old children and set specific cut-off points for MetS at 75th percentile for girls and 90th percentile for boys. Alías-Hernández et al. [52] similarly identified HOMA-IR levels above the 90th percentile as high-risk indicators for metabolic complications in 2–10 years old children. Significant discrepancies were observed between the study-specific 50th and 90th percentiles and the IDEFICS reference percentiles (Fig. 4). Separately, three studies reported HbA1c percentile distributions which showed substantial inter-study variation based on age and sex (Fig. 6). Notably, these studies failed to provide defined cut-offs for identifying individuals with elevated risk [48, 49, 51]. [Figure 6]. The lack of publicly available HbA1c reference values from IDEFICS study did not allow to reconstruct the reference curves for comparison. For fasting glucose, only one study provided constructed percentiles [49]; however, that study did not propose a specific cut-off value to identify high-risk individuals.

#### Biomarkers for Lipid Metabolism

**Fig. 7:**
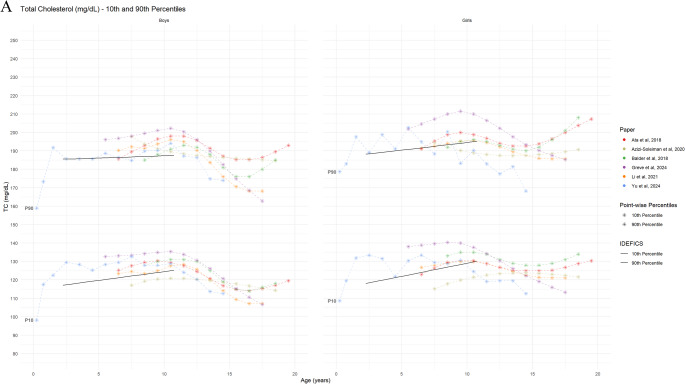

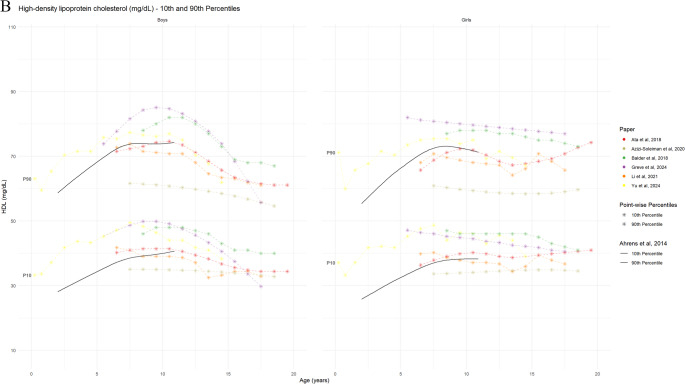

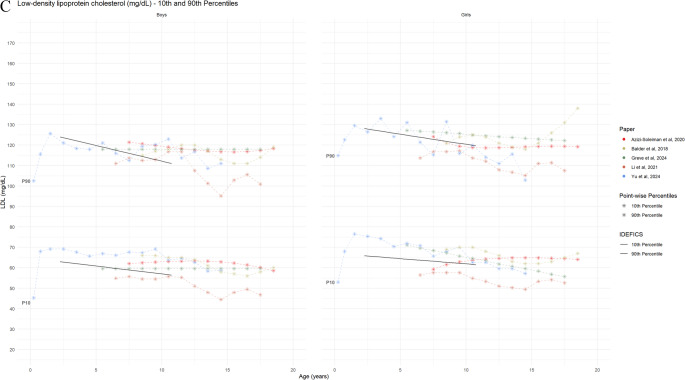
Reference values for lipid parameters by age and sex (absolute values) Panel 7A: 10th and 90th percentile curves of the TC from the included studies Panel 7B: 10th and 90th percentile curves of HDL-C from the included studies Panel 7C: 2.5th, 3rd and/or 97th/97.5th percentile curves of LDL-C, from the included studies The black lines show 10th and 90th percentiles reference from Ahrens et al., 2014 [5]

Eight studies provided reference curves for lipid profiles, including TC (8 studies), LDL-C (6 studies), and HDL-C (8 studies). A comparison of the TC reference curves demonstrated good overall agreement for age- and sex-specific 50th and 90th percentiles (Figure S4, Fig. 7 Panel A). While slight variations were noted in the age- and sex-specific HDL-C percentiles across studies, the LDL-C reference values were comparable among the three studies that reported them. Three studies utilized these percentiles to present specific diagnostic criteria for TC, LDL-C, TG, and HDL-C [55, 57, 63] or cut-off points [54, 64], while another three studies did not mention any diagnostic criteria [51, 56, 58]. Dyslipidaemia definitions varied across the included studies, utilizing different thresholds and diagnostic criteria. The overlay plot analysis revealed minimal variation among studies for the 10th and 90th percentiles of TC, LDL-C, and HDL-C. However, due to inherent differences in age stratification, the IDEFICS definition of dyslipidaemia was not directly comparable to other studies’ lipid reference values, particularly in children under 6 or 7 years of age. For children aged 7–11 years, the 90th percentile of the lipid profile biomarkers from the included studies generally clustered around the corresponding 90th reference percentile established by the IDEFICS definition, with the exception of TC.

### Risk of Bias Assessment

The results of the risk of bias assessment for all included studies are presented in detail in Table S2. Overall, the evidence base was found to have a high level of internal validity, with the vast majority of the 46 included studies rated as having high to moderate quality. Specifically, 35 studies were classified as high quality (scoring 18–20 points), and 10 studies were identified as moderate-to-high quality (15–17 points). Only one study received a score of 14 points, indicating the lowest quality within the included literature.

## Discussion

This systematic review provides a comprehensive summary of the recent evidence regarding reference curves for MetS components in paediatric populations. Based on data from 46 studies, notable variations were observed in the reference curves and definitions of abdominal obesity based on waist circumference and impaired glucose metabolism, both across studies and in comparison, to existing definition based on data of the IDEFICS study, comparatively smaller variations were observed for hypertension and dyslipidaemia (TC and HDL-C only) from the existing definitions. Results from this review revealed limited data availability for young age-group (0–4 years), as well as substantial heterogeneity in study methodologies including differences in study design, assays and statistical approaches used to derive reference curves. These findings underscore the need for standardized protocols, with data from covering diverse populations to develop harmonized reference curves for consistent definition of MetS and its components.

Majority of the included studies proposed reference curves on waist circumference, emphasizing its role as an important indicator for abdominal obesity and subsequent risk of chronic diseases. Despite this acknowledgement, varying percentile cut-offs were used to define elevated risk complicating clinical interpretation. Nevertheless, most of the studies adopted the 90th percentile as cut-off for abdominal obesity, which is in line with existing evidence [20, 21] that WC above 90th percentile is associated higher risk of developing cardiovascular diseases in later life. Nonetheless, large variations were observed in 90th percentile of WC across studies, which could be attributed to both the methodological heterogeneity in studies and biological variation (e.g. differences in fat distribution by country or ethnicity). These differences undermine direct comparison between studies and highlight importance of standardized protocols in development of reference curves, to ensure a reliable definition of abdominal obesity in children and adolescents.

The evidence for glucose metabolism markers remains limited. Only small number of studies reported reference curves for biomarkers of glucose metabolism and a corresponding definition of impaired glucose metabolism. Large heterogeneity across studies, including age-specific differences in study samples makes the direct comparison difficult. However, it could point to the methodological and logistical challenges of assessing these biomarkers in paediatric populations especially in children 0–5 years. Still, it is of utmost importance to increase efforts in collecting markers of glucose metabolism in this age group. Previous research has shown that physiological transient insulin resistance develops in children during puberty and decreases again until adulthood, regardless of obesity [65]. The decline in insulin sensitivity during the pubertal period is believed to result in an increase in glucose-stimulated insulin secretion [66]. Given the rising prevalence of type 2 diabetes and the corresponding risk factors, it is important to establish harmonized definition of impaired glucose metabolism in a sample comprising large diverse populations using standardized protocols.

In this review, comparatively consistent reference curves especially 90th and 95th percentile of DBP for some studies were identified that aligned with the established standard IDEFICS study definition [5]. Despite methodological differences in the studies, this consistency for some studies may reflect stricter protocols and/or smaller measurement variations. Similarly, comparatively consistent median (50th) and higher (90th) percentiles were observed for reference curves of TC, HDL-C, and LDL-C. However, differences exist in the definition of lipidemic as some studies used fixed cut-offs whereas others relied on percentiles. Normal cholesterol concentrations vary with age and sex; therefore, fixed cut-offs may under- or over-estimate dyslipidaemia in children [67].

Methods to derive references curves also varied across studies. Studies employed LMS, GAMLSS or other regression approach for reference curves. Among these methods, simple linear regression is not able to capture the skewness of the distributions or non-linear age dependencies. The difference in statistical approaches on modelling the reference curves highlights the need for standardized approaches [68], since these differences can introduce perceived difference in estimated reference values and diagnostic thresholds among different populations. For this review, we chose reference curves proposed by researchers of the IDEFICS study [5] for reference because this is based on internationally derived, harmonized paediatric reference curves based on large multi-center cohort.

Although, the included studies in this review came from large number of countries spanning four continents, representation of studies with biomarkers were significantly low for middle- and lower-income countries (LMIC), especially from Africa. The low availability of data, especially the blood-based biomarker data, from LMIC is a particular concern, given the high proportion of infants, children, and adolescents suffering from stunting, acute malnutrition and micronutrient deficiencies. Many LMIC today are undergoing a coexistence of the double burden of underweight and overweight/obesity exposing these population to the risk of metabolic complications. This is a significant gap in evidence as the overall paediatric population and burden of metabolic disorders is already higher in many of these countries [69, 70]. Lack of studies from these regions preclude the possibility of developing region or ethnicity specific reference curves.

The strengths of this review are the large number of studies included, covering a wide range of countries and regions. This diversity provides a more comprehensive understanding of paediatric health indicators on a global level, offering useful insights into variations in different populations. Moreover, we focused on recently published literature to capture the recent development in the field. However, there are also some limitations. One important limitation is the range of publication years covered by the review since important findings published in years prior to 2018 were not included. We used the BIOCROSS tool to evaluate the quality/risk of bias in the included studies [17]. However, the tool may not be suitable for studies that reuse data. For example, Hu et al. [49] used National Health and Nutrition Examination Survey (NHANES) data for their analysis but did not provide brief description of the study methodology with reference and was subsequently rated as having low quality, using this scale Nevertheless, it should be noted that the overall strength of the evidence base was consistently high, with 45 of the 46 included studies demonstrating high to moderate internal validity. Thus, the potential misclassification of a single secondary analysis study’s quality does not appear to compromise the robust nature of the synthesized findings.Furthermore, the review highlights significant gaps in the evidence base, specifically a lack of data from middle- and lower-income countries and limited biomarker information for children aged 0–4 years. These findings are further complicated by substantial methodological heterogeneity, as variations in study designs, laboratory assays, and statistical approaches—combined with inconsistent percentile cut-offs—hinder direct comparisons and underscore the urgent need for standardized assessment protocols across diverse global populations.

## Conclusion

This review identified large variability in the reference curves for defining abdominal obesity and impaired glucose metabolism in paediatric populations. In contrast, the indicators denoting early-life hypertension and dyslipidaemia showed comparatively better consistency with existing definitions, though inter-study variation exists. The variation and inconsistent definitions of various MetS components underscore the need for harmonized evidence-based definition based on a sample of a diverse population. Such reference curves would be key for an early and accurate diagnosis and effective intervention planning for clinicians and health practitioners.

## Supplementary Information

Below is the link to the electronic supplementary material.

Supplementary Material 1 (DOCX.666 KB)

## Data Availability

No datasets were generated or analysed during the current study.

## Key References

- Reisinger C, Nkeh-Chungag BN, Fredriksen PM, Goswami N. The prevalence of pediatric metabolic syndrome-a critical look on the discrepancies between definitions and its clinical importance. Int J Obes (Lond). 2021;45(1):12–24. 10.1038/s41366-020-00713-1.

A critical review of pediatric metabolic syndrome highlighting discrepancies between different definitions and their clinical significance.

- Ahrens W, Moreno LA, Marild S, Molnar D, Siani A, De Henauw S, Bohmann J, et al. Metabolic syndrome in young children: definitions and results of the IDEFICS study. Int J Obes (Lond). 2014;38 Suppl 2(Suppl 2): S4-14.10.1038/ijo.2014.130.

Based on data from a large European cohort, the study proposes a specific set of criteria for young children aged 2 to 11 years, based on components like waist circumference, triglycerides, HDL-C, blood pressure, and glucose or insulin levels, detailing the prevalence of MetS in this age group.

- Wirsching J, Graßmann S, Eichelmann F, Harms LM, Schenk M, Barth E, et al. Development and reliability assessment of a new quality appraisal tool for cross-sectional studies using biomarker data (BIOCROSS). BMC Medical Research Methodology. 2018;18(1):122..10.1186/s12874-018-0583-x.

A quality appraisal tool specifically designed for cross-sectional studies that use biomarker data. This tool aims to systematically evaluate the quality of such studies.

- Wasniewska M, Pepe G, Aversa T, Bellone S, de Sanctis L, Di Bonito P, Faienza MF, et al. Skeptical Look at the Clinical Implication of Metabolic Syndrome in Childhood Obesity. Children (Basel). 2023;10(4). 10.3390/children10040735.

A review that highlights the lack of accepted definition of metabolic syndrome in children and emphasise the need to focus on early detection and prevention of obesity and related complications.

## Abbreviations

DBP: Diastolic blood pressure
HbA1c: Hemoglobin A1c
HDL-C: High Density Lipoprotein Cholesterol
HOMA-IR: homeostasis model assessment of insulin resistance
GAMLSS: Generalized Additive Models for Location, Scale, and Shape
HTN: hypertension
LMS: Lambda-Mu-Sigma: LDL-C: Low Density Lipoprotein Cholesterol
ND: Not described
SBP: Systolic blood pressure
TC: Total cholesterol
TG: Triglycerides
WC: Waist circumference
