# Supplementary material for: Reference Curves for Metabolic Syndrome Indicators in Children and Adolescents: A Global Systematic Review
Source: Curr Obes Rep. 2026 Jan 5;15(1):3. doi: 10.1007/s13679-025-00679-z (PMC12769977; doi:10.1007/s13679-025-00679-z)

**Supplementary Table**

## Table S1: Search strategies implemented in different electronic databases

| PUBMED search strategy | | | |
| --- | --- | --- | --- |
| # | searches | | |
| 1 | "newborn*"[Title/Abstract] OR "infant*"[Title/Abstract] OR "neonate*"[Title/Abstract] OR "baby"[Title/Abstract] ΟR "babies"[Title/Abstract] OR "toddler*"[Title/Abstract] OR "child*"[Title/Abstract] OR "schoolboy*"[Title/Abstract] OR "schoolgirl*"[Title/Abstract] OR "adolescen*"[Title/Abstract] OR "juvenile*"[Title/Abstract] OR "teenager*"[Title/Abstract] OR "teen*"[Title/Abstract] OR "youth*"[Title/Abstract] OR "pre-adult*"[Title/Abstract] OR "pediatric*"[Title/Abstract] OR "paediatric*"[Title/Abstract] | | |
| 2 | "infant"[Mesh Terms] OR "child" [Mesh Terms] OR "adolescent" [Mesh Terms] | | |
| 3 | "Biomarker*"[Title/Abstract] OR "marker*"[Title/Abstract] OR "indicator*"[Title/Abstract] OR "Serum Marker*"[Title/Abstract] OR "Waist circumference*"[Title/Abstract] OR "Waist measurement*"[Title/Abstract] OR "Triglyceride*"[Title/Abstract] OR "TG"[Title/Abstract] OR "triacylglycerol"[Title/Abstract] OR "triacylglyceride*"[Title/Abstract] OR "High-density lipoprotein*"[Title/Abstract] OR "HDL*"[Title/Abstract] OR "Blood Glucose"[Title/Abstract] OR "Glucose"[Title/Abstract] OR "blood Sugar*"[Title/Abstract] OR "systolic blood pressure"[Title/Abstract] OR "SBP"[Title/Abstract] OR "diastolic blood Pressure"[Title/Abstract] OR "DBP"[Title/Abstract] OR "Homeostasis Model Assessment"[Title/Abstract] OR "insulin resistance"[Title/Abstract] OR "HOMA-IR"[Title/Abstract] OR "glycated hemoglobin*"[Title/Abstract] OR "Hba1c"[Title/Abstract] OR "C-peptide"[Title/Abstract] OR "metabolic Syndrome"[Title/Abstract] OR "Dysmetabolic syndrome X"[Title/Abstract] OR "MetS"[Title/Abstract] | | |
| 4 | "Biomarkers" [Mesh Terms] OR "Waist circumference" [Mesh Terms] OR "Triglycerides" [Mesh Terms] OR "Lipoproteins, HDL"[Mesh Terms] OR "Blood Glucose" [Mesh Terms] OR "blood pressure" [Mesh Terms] OR "metabolic Syndrome" [Mesh Terms] | | |
| 5 | (("reference"[Title/Abstract]) AND ("value*"[Title/Abstract] OR "interval*"[Title/Abstract] OR "range*"[Title/Abstract] OR "limit*"[Title/Abstract] OR "curve*"[Title/Abstract] OR "chart*"[Title/Abstract])) OR ("centile curve*"[Title/Abstract]) | | |
| 6 | "reference values"[MeSH Terms] | | |
| 7 | #1 OR #2 | | |
| 8 | #3 OR #4 | | |
| 9 | #5 OR #6 | | |
| 10 | #7 AND #8 AND #9 | | |
| 11 | #7 AND #8 AND #9 Filters: from 2018-2025 | | |
| Scopus search strategy | | |  |
| # | | searches |  |
| 1 | TITLE-ABS ("newborn*" OR "infant*" OR "neonate*" OR "baby" OR "babies" OR "toddler*" OR "child*" OR "schoolboy*" OR "schoolgirl*" OR "adolescen*" OR "juvenile*" OR "teenager*" OR "teen*" OR "youth*" OR "pre adult*" OR "pediatric*" OR "paediatric*") | |  |
| 2 | TITLE-ABS("Biomarker*" OR "marker*" OR "indicator*" OR "Serum Marker*" OR "Waist circumference*" OR "Waist measurement*" OR "Triglyceride*" OR "TG" OR "triacylglycerol" OR "triacylglyceride*" OR "High-density lipoprotein*" OR "HDL*" OR "Blood Glucose" OR "Glucose" OR "blood Sugar*" OR "systolic blood pressure" OR "SBP" OR "diastolic blood Pressure" OR "DBP" OR "Homeostasis Model Assessment" OR "insulin resistance" OR "HOMA-IR" OR "glycated hemoglobin*" OR "Hba1c" OR "C-peptide" OR "metabolic Syndrome" OR "Dysmetabolic syndrome X" OR "MetS") | |  |
| 3 | TITLE-ABS(("reference") AND ("value*" OR "interval*" OR "range*" OR "limit*" OR "curve*" OR "chart*") OR ("centile curve*")) | |  |
| 4 | #1 AND #2 AND #3 | |  |
| 5 | #1 AND #2 AND #3 AND PUBYEAR > 2017 AND PUBYEAR < 2026 | |  |
| Web of science search strategy | | | |
| # | | Searches | |
| 1 | | TI=(("newborn*" OR "infant*" OR "neonate*" OR "baby" OR "babies" OR "toddler*" OR "child*" OR "schoolboy*" OR "schoolgirl*" OR "adolescen*" OR "juvenile*" OR "teenager*" OR "teen*" OR "youth*" OR "pre adult*" OR "pediatric*" OR "paediatric*")) OR AB=(("newborn*" OR "infant*" OR "neonate*" OR "baby" OR "babies" OR "toddler*" OR "child*" OR "schoolboy*" OR "schoolgirl*" OR "adolescen*" OR "juvenile*" OR "teenager*" OR "teen*" OR "youth*" OR "pre adult*" OR "pediatric*" OR "paediatric*")) | |
| 2 | | TI=(("Biomarker*" OR "marker*" OR "indicator*" OR "Serum Marker*" OR "Waist circumference*" OR "Waist measurement*" OR "Triglyceride*" OR "TG" OR "triacylglycerol" OR "triacylglyceride*" OR "High-density lipoprotein*" OR "HDL*" OR "Blood Glucose" OR "Glucose" OR "blood Sugar*" OR "systolic blood pressure" OR "SBP" OR "diastolic blood Pressure" OR "DBP" OR "Homeostasis Model Assessment" OR "insulin resistance" OR "HOMA-IR" OR "glycated hemoglobin*" OR "Hba1c" OR "C-peptide" OR "metabolic Syndrome" OR "Dysmetabolic syndrome X" OR "MetS" )) OR AB=(("Biomarker*" OR "marker*" OR "indicator*" OR "Serum Marker*" OR "Waist circumference*" OR "Waist measurement*" OR "Triglyceride*" OR "TG" OR "triacylglycerol" OR "triacylglyceride*" OR "High-density lipoprotein*" OR "HDL*" OR "Blood Glucose" OR "Glucose" OR "blood Sugar*" OR "systolic blood pressure" OR "SBP" OR "diastolic blood Pressure" OR "DBP" OR "Homeostasis Model Assessment" OR "insulin resistance" OR "HOMA-IR" OR "glycated hemoglobin*" OR "Hba1c" OR "C-peptide" OR "metabolic Syndrome" OR "Dysmetabolic syndrome X" OR "MetS")) | |
| 3 | | TI=((("reference") AND ("value*" OR "interval*" OR "range*" OR "limit*" OR "curve*" OR "chart*") OR ("centile curve*"))) OR AB=((("reference") AND ("value*" OR "interval*" OR "range*" OR "limit*" OR "curve*" OR "chart*") OR ("centile curve*"))) | |
| 4 | | #1 AND #2 AND #3 | |
| 5 | | #1 AND #2 AND #3 AND Publication Years (2025 OR 2024 OR 2023 OR 2022 OR 2021 OR 2020 OR 2019 OR 2018) | |

Table S2: Quality assessment scores* of the included studies using the BIOCROSS tool

| SNo | **Year/Author** | **Item 1** | **Item 2** | **Item 3** | **Item 4** | **Item 5** | **Item 6** | **Item 7** | **Item 8** | **Item 9** | **Item 10** | **Final Score** |
| --- | --- | --- | --- | --- | --- | --- | --- | --- | --- | --- | --- | --- |
| 1 | Ahmadi et al (2020) | 2 | 2 | 2 | 2 | 1 | 2 | 2 | 2 | 2 | 1 | 18 |
| 2 | Alías-Hernández et al (2018) | 1 | 2 | 2 | 2 | 2 | 2 | 2 | 2 | 2 | 1 | 18 |
| 3 | AlSalloum et al (2020) | 1 | 1 | 2 | 2 | 2 | 2 | 0 | 2 | 2 | 1 | 15 |
| 4 | Andaki et al (2018) | 2 | 1 | 2 | 2 | 2 | 2 | 2 | 2 | 2 | 0 | 17 |
| 5 | Asif et al (2020) | 2 | 2 | 2 | 2 | 2 | 2 | 1 | 2 | 2 | 2 | 19 |
| 6 | Ata et el (2018) | 1 | 2 | 2 | 2 | 2 | 2 | 2 | 2 | 2 | 2 | 19 |
| 7 | Azizi-Soleiman et al (2020) | 1 | 2 | 2 | 2 | 2 | 2 | 2 | 2 | 1 | 0 | 16 |
| 8 | Balder et al, 2018 | 2 | 2 | 2 | 2 | 2 | 2 | 2 | 2 | 2 | 2 | 20 |
| 9 | Bojanic et al (2020) | 2 | 2 | 2 | 2 | 2 | 1 | 2 | 2 | 2 | 2 | 19 |
| 10 | Chissini 2020 | 2 | 2 | 2 | 2 | 2 | 2 | 2 | 2 | 2 | 2 | 20 |
| 11 | Cossio-Bolaños et al (2020) | 2 | 2 | 2 | 2 | 2 | 2 | 2 | 2 | 2 | 2 | 20 |
| 12 | El-Shafie et al (2018) | 2 | 2 | 2 | 2 | 2 | 2 | 2 | 2 | 2 | 2 | 20 |
| 13 | Fredriksen et al 2018 | 2 | 1 | 2 | 2 | 2 | 2 | 2 | 2 | 2 | 2 | 19 |
| 14 | Fujita et al (2023) | 1 | 2 | 2 | 2 | 2 | 2 | 2 | 2 | 0 | 2 | 17 |
| 15 | Ghouili et al (2020) | 2 | 2 | 2 | 2 | 2 | 2 | 2 | 2 | 1 | 0 | 17 |
| 16 | Gomez-Campos et al (2019) | 2 | 1 | 1 | 2 | 2 | 2 | 2 | 2 | 2 | 2 | 18 |
| 17 | Hasegawa et al (2021) | 1 | 1 | 2 | 2 | 2 | 2 | 0 | 2 | 1 | 2 | 15 |
| 18 | Hovestadt et al (2022) | 2 | 2 | 2 | 2 | 2 | 2 | 2 | 2 | 2 | 2 | 20 |
| 19 | Hu et al (2021) | 1 | 1 | 2 | 2 | 2 | 1 | 2 | 1 | 1 | 2 | 15 |
| 20 | Jardim et al (2020) | 2 | 1 | 2 | 2 | 2 | 2 | 2 | 2 | 2 | 2 | 19 |
| 21 | Jáuregui-Ulloa et al (2021) | 2 | 2 | 2 | 2 | 2 | 2 | 2 | 2 | 1 | 2 | 19 |
| 22 | Karki et al (2018) | 2 | 1 | 2 | 2 | 2 | 2 | 2 | 2 | 2 | 2 | 19 |
| 23 | Keskinoglu et al (2020) | 1 | 2 | 2 | 2 | 2 | 2 | 2 | 2 | 2 | 1 | 18 |
| 24 | Kim et al, 2019 | 2 | 2 | 2 | 2 | 2 | 2 | 2 | 2 | 0 | 2 | 18 |
| 25 | Kulaga et al, 2023 | 2 | 2 | 2 | 2 | 2 | 2 | 2 | 2 | 0 | 2 | 18 |
| 26 | Lee et al, 2020 | 2 | 2 | 2 | 2 | 2 | 2 | 2 | 2 | 2 | 2 | 20 |
| 27 | Lee et al, 2022 | 2 | 1 | 2 | 2 | 2 | 2 | 2 | 2 | 2 | 2 | 19 |
| 28 | Li et al, 2021 | 2 | 2 | 2 | 2 | 2 | 2 | 2 | 2 | 2 | 1 | 19 |
| 29 | Marrodan Serrano et al, 2021 | 2 | 2 | 2 | 2 | 2 | 2 | 2 | 2 | 0 | 0 | 16 |
| 30 | Montazeri- Najafabady et al, 2023 | 2 | 2 | 2 | 2 | 2 | 2 | 2 | 2 | 2 | 1 | 19 |
| 31 | Muyumba et al, 2018 | 2 | 1 | 2 | 2 | 2 | 2 | 2 | 2 | 2 | 2 | 19 |
| 32 | Ramgopal et al, 2023 | 2 | 2 | 2 | 2 | 2 | 2 | 2 | 1 | 1 | 2 | 18 |
| 33 | Sarna et al, 2021 | 1 | 2 | 2 | 2 | 2 | 2 | 2 | 2 | 1 | 2 | 18 |
| 34 | Shah et al, 2020 | 2 | 1 | 2 | 2 | 2 | 2 | 2 | 2 | 2 | 0 | 17 |
| 35 | Sooriyakanthan et al, 2018 | 2 | 2 | 2 | 2 | 2 | 2 | 0 | 2 | 0 | 2 | 16 |
| 36 | Sousa et al, 2019 | 2 | 2 | 2 | 2 | 2 | 2 | 0 | 2 | 2 | 0 | 16 |
| 37 | Thangjam et al, 2018 | 2 | 2 | 2 | 2 | 2 | 2 | 2 | 2 | 0 | 2 | 18 |
| 38 | Van Eyck et al, 2021 | 2 | 1 | 2 | 2 | 2 | 2 | 2 | 2 | 2 | 2 | 19 |
| 39 | Vendula et al, 2021 | 2 | 1 | 2 | 2 | 2 | 2 | 2 | 2 | 2 | 2 | 19 |
| 40 | Xiao et al, 2019 | 2 | 2 | 2 | 2 | 2 | 2 | 2 | 2 | 2 | 2 | 20 |
| 41 | Zong et al, 2018 | 2 | 2 | 2 | 2 | 2 | 2 | 2 | 2 | 1 | 2 | 19 |
| 42 | Gromnatska, 2024 | 2 | 1 | 1 | 2 | 1 | 2 | 2 | 2 | 0 | 2 | 15 |
| 43 | Alves Junior et al, 2024 | 2 | 2 | 2 | 2 | 2 | 2 | 2 | 2 | 2 | 2 | 20 |
| 44 | Ren et al, 2024 | 2 | 2 | 2 | 2 | 2 | 2 | 2 | 1 | 2 | 0 | 17 |
| 45 | Greve et al, 2024 | 2 | 1 | 2 | 2 | 2 | 2 | 2 | 2 | 2 | 2 | 19 |
| 46 | Yu et al, 2024 | 2 | 2 | 2 | 2 | 2 | 2 | 2 | 2 | 0 | 2 | 18 |

| Item 1 | Hypothesis/Objective |
| --- | --- |
| Item 2 | Study population selection |
| Item 3 | Study population representativeness |
| Item 4 | Study population characteristics |
| Item 5 | Statistical analyses |
| Item 6 | Interpretation and evaluation of results |
| Item 7 | Study limitations |
| Item 8 | Specimen characteristics and assay methods |
| Item 9 | Laboratory measurement |
| Item 10 | Biomarker data modeling |

*Score: 0=no information provided about the item; 1=some information provided about the item; 2=complete information provided about the item.

Figure S1: Proportion of included studies reporting age- and sex-specific reference curves by age groups (N = 46).


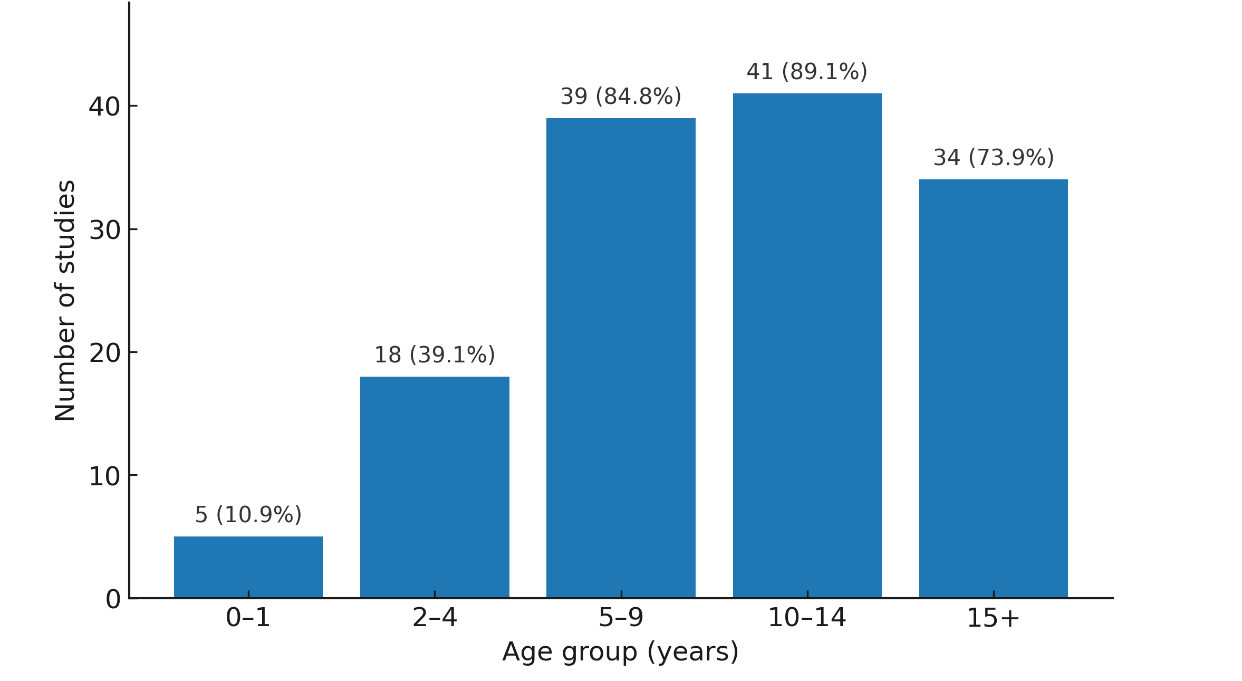


Percentages do not sum to 100%, as some studies covered multiple age groups.

Figure S2: Reference values for waist circumference by age and sex (absolute values)
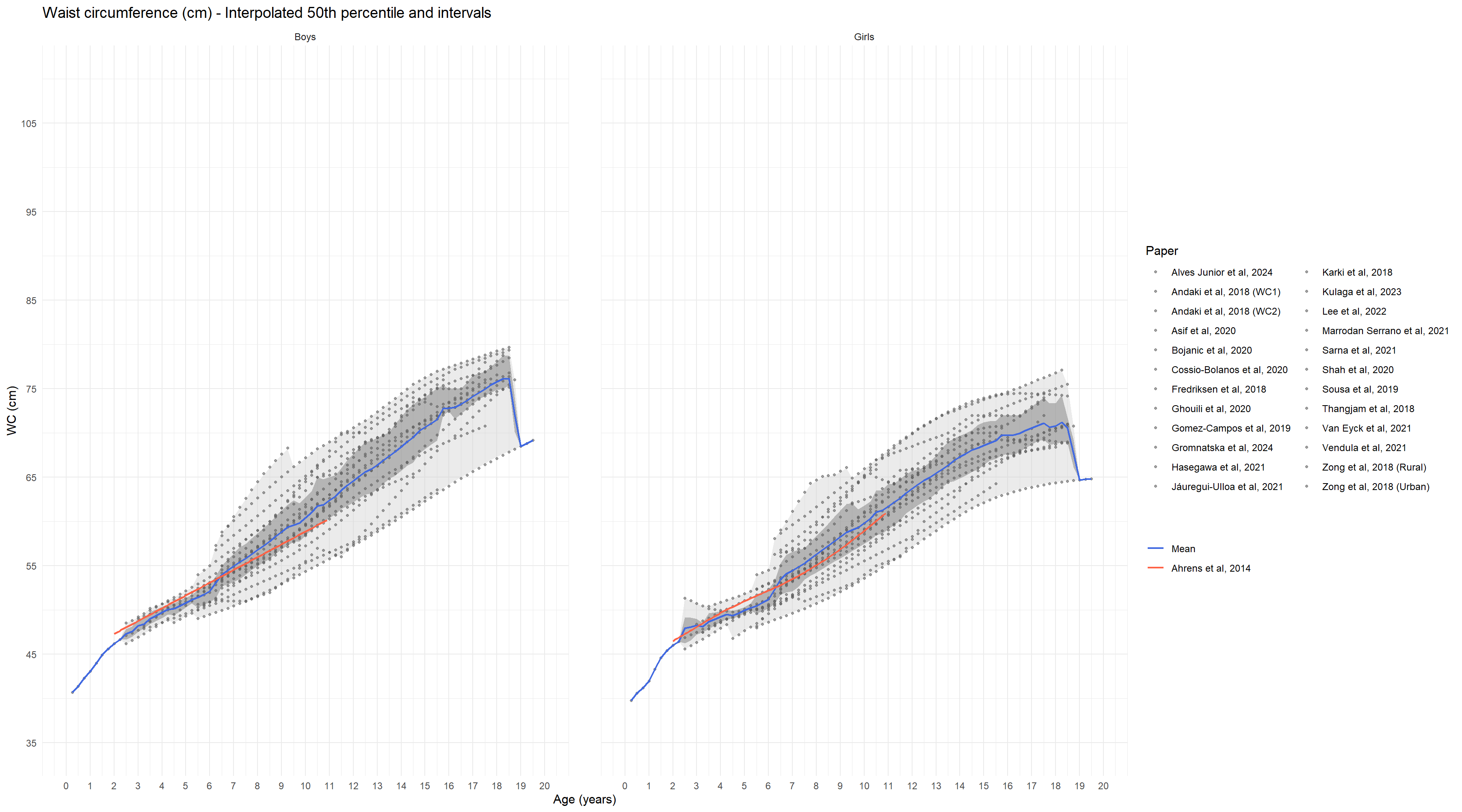


50^th^ percentile curves of included studies, corresponding intervals bands covering all studies (light grey) and 50% of studies (dark grey) and mean 50^th^ percentile

The red line shows 90th percentile reference from IDEFICS definition by Ahrens et al, 2014

Figure S3: Reference values for systolic (Panel A) and diastolic blood pressure (Panel B) by age and sex (absolute values).


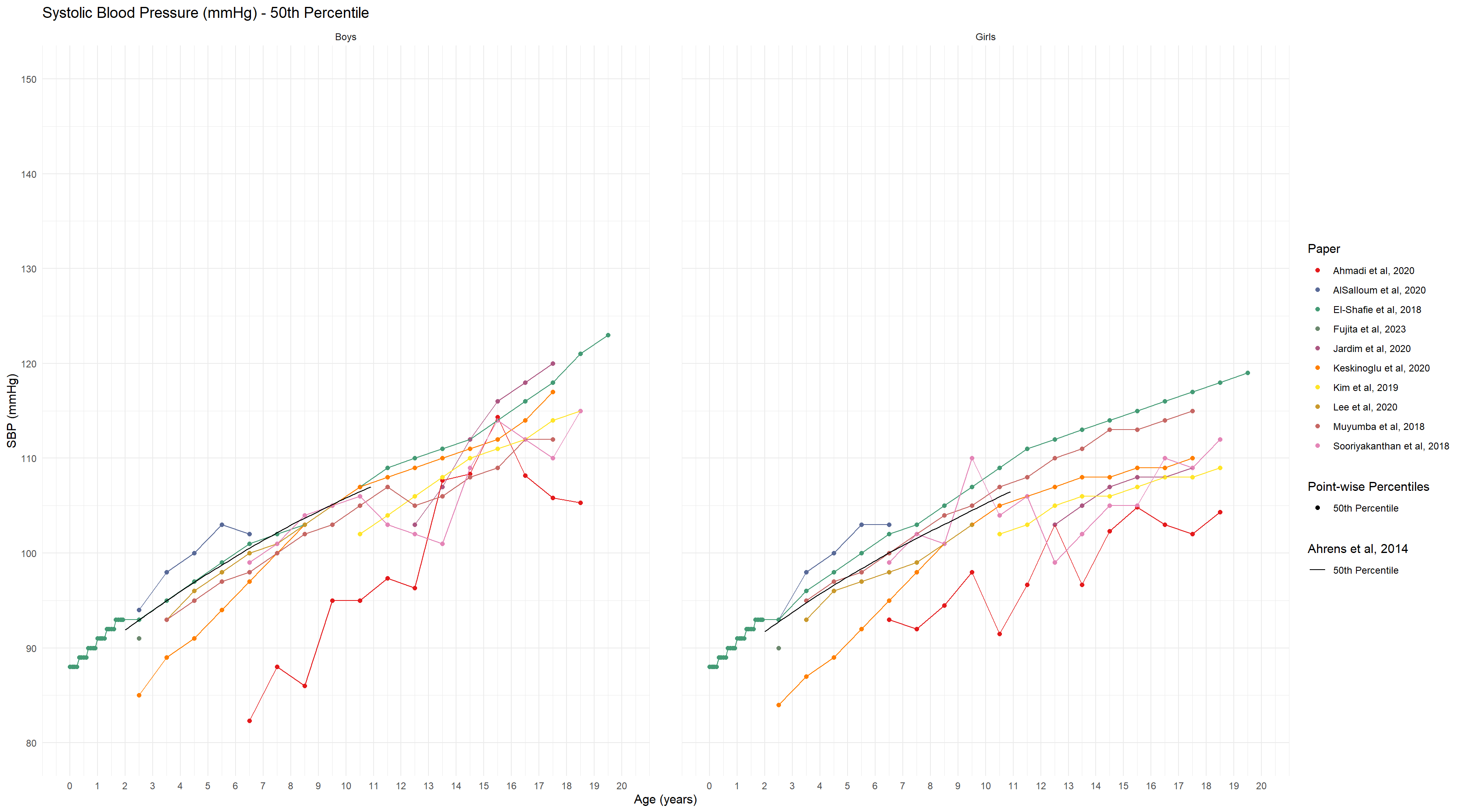

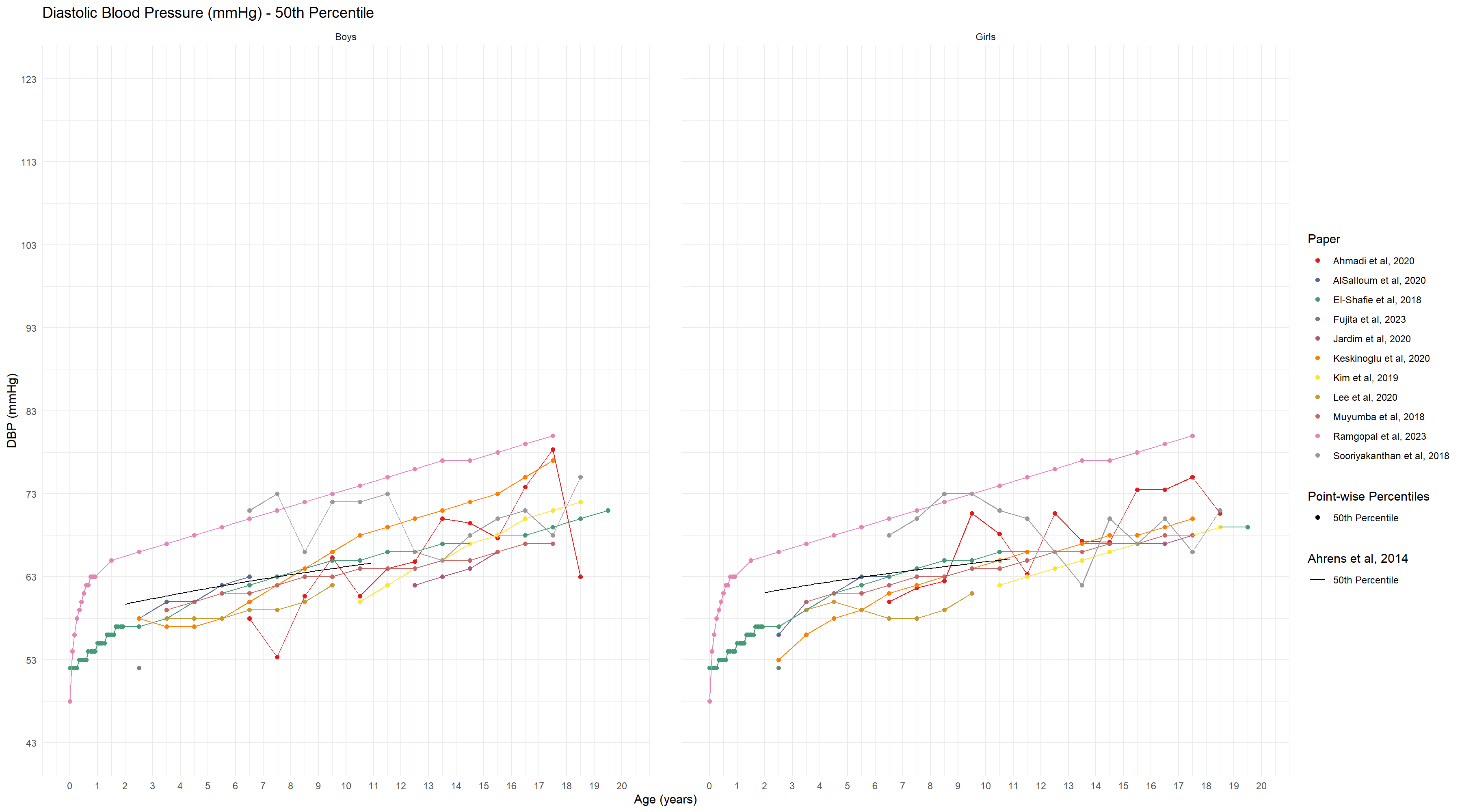


The black line shows 50^th^ percentile reference from IDEFICS definition

Figure S4: 50^th^ percentile curves of the lipid profile from the included studies.

Panel A: Total Cholesterol


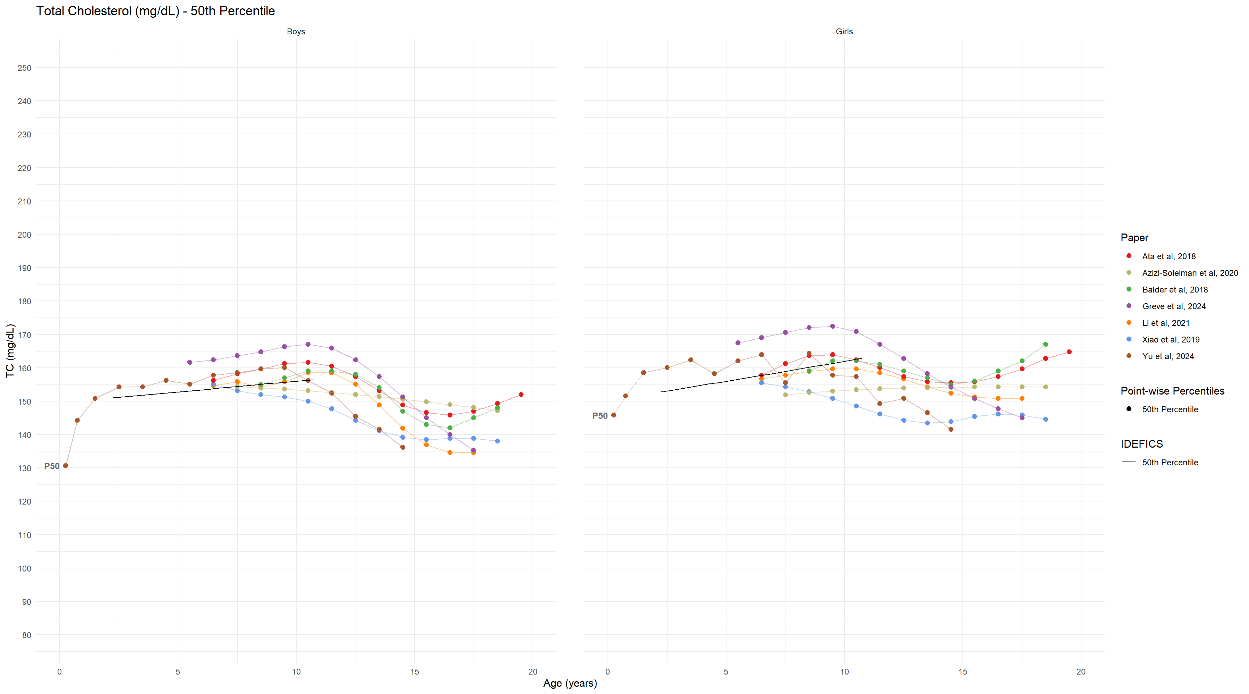


Panel B: HDL


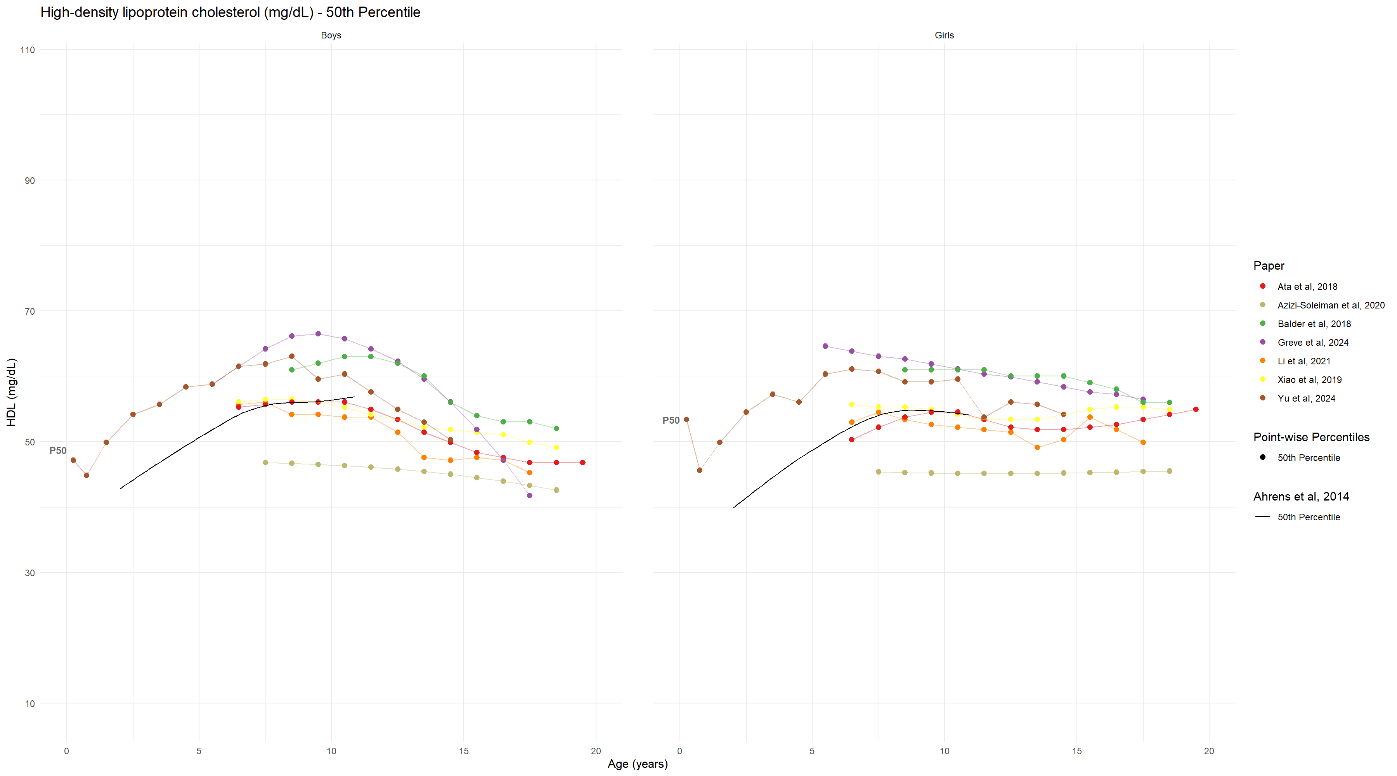


Panel C: LDL


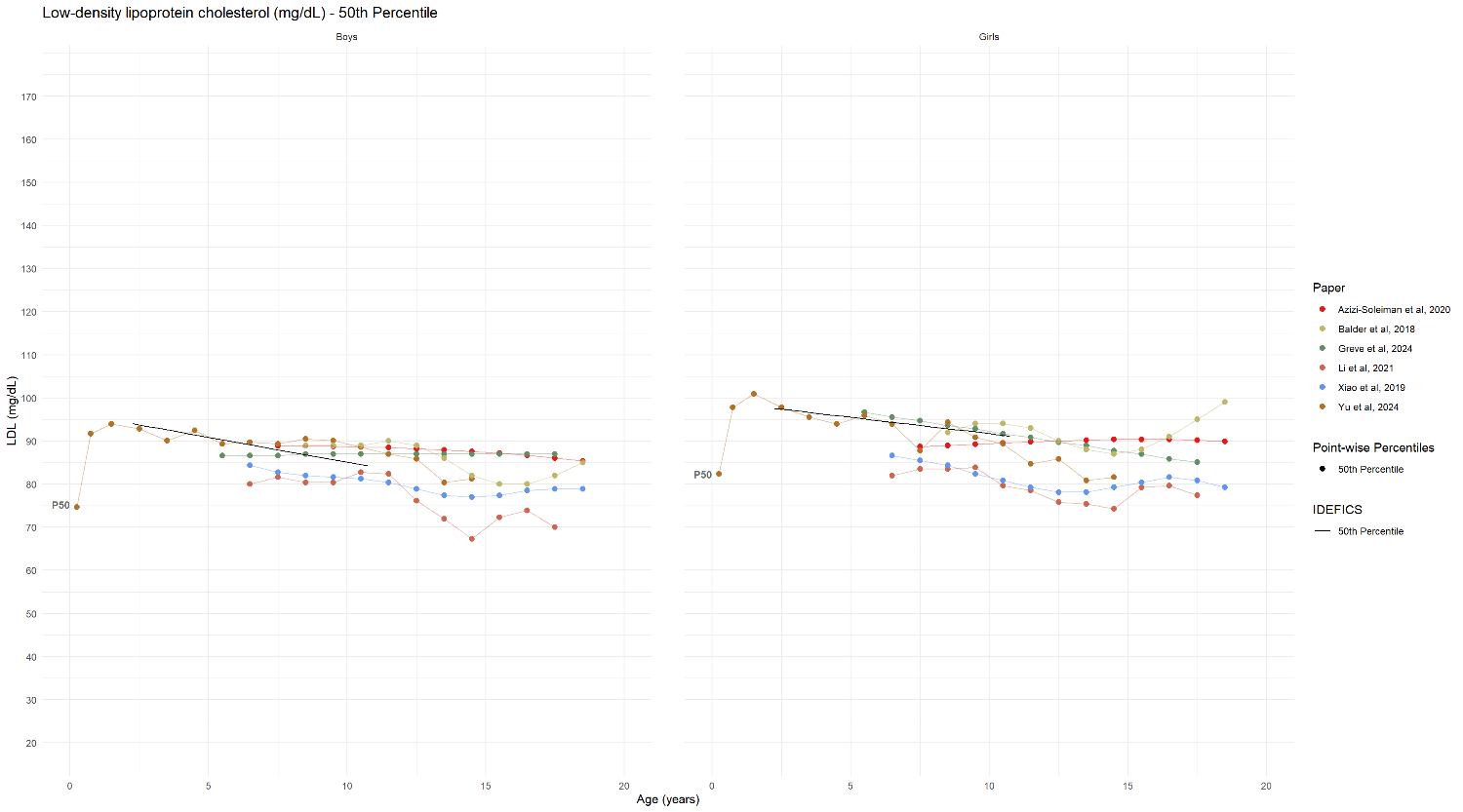

Supplement: Supplementary file 1 — (DOCX.666 KB) [file 13679_2025_679_MOESM1_ESM.docx]
